# Association of self-identified race and genetic ancestry with the immunogenomic landscape of primary prostate cancer

**DOI:** 10.1172/jci.insight.162409

**Published:** 2023-02-08

**Authors:** Thiago Vidotto, Eddie L. Imada, Farzana Faisal, Sanjana Murali, Adrianna A. Mendes, Harsimar Kaur, Siqun Zheng, Jianfeng Xu, Edward M. Schaeffer, William B. Isaacs, Karen S. Sfanos, Luigi Marchionni, Tamara L. Lotan

**Affiliations:** 1 Department of Pathology, Johns Hopkins University School of Medicine, Baltimore, Maryland, USA.; 2 Department of Pathology, Weill-Cornell School of Medicine, New York, New York, USA.; 3 Program for Personalized Cancer Care, NorthShore University Health System, Evanston, Illinois, USA.; 4 Department of Urology, Northwestern University School of Medicine, Chicago, Illinois, USA.; 5 Department of Urology and; 6 Department of Oncology, Johns Hopkins University School of Medicine, Baltimore, Maryland, USA.

**Keywords:** Genetics, Cellular immune response, Genetic variation, Prostate cancer

## Abstract

The genomic and immune landscapes of prostate cancer differ by self-identified race. However, few studies have examined the genome-wide copy number landscape and immune content of matched cohorts with genetic ancestry data and clinical outcomes. Here, we assessed prostate cancer somatic copy number alterations (sCNA) and tumor immune content of a grade-matched, surgically treated cohort of 145 self-identified Black (BL) and 145 self-identified White (WH) patients with genetic ancestry estimation. A generalized linear model adjusted with age, preoperative prostate-specific antigen (PSA), and Gleason Grade Group and filtered for germline copy number variations (gCNV) identified 143 loci where copy number varied significantly by percent African ancestry, clustering on chromosomes 6p, 10q, 11p, 12p, and 17p. Multivariable Cox regression models adjusted for age, preoperative PSA levels, and Gleason Grade Group revealed that chromosome 8q gains (including *MYC*) were significantly associated with biochemical recurrence and metastasis, independent of genetic ancestry. Finally, Treg density in BL and WH patients was significantly correlated with percent genome altered, and these findings were validated in the TCGA cohort. Taken together, our findings identify specific sCNA linked to genetic ancestry and outcome in primary prostate cancer and demonstrate that Treg infiltration varies by global sCNA burden in primary disease.

## Graphical abstract

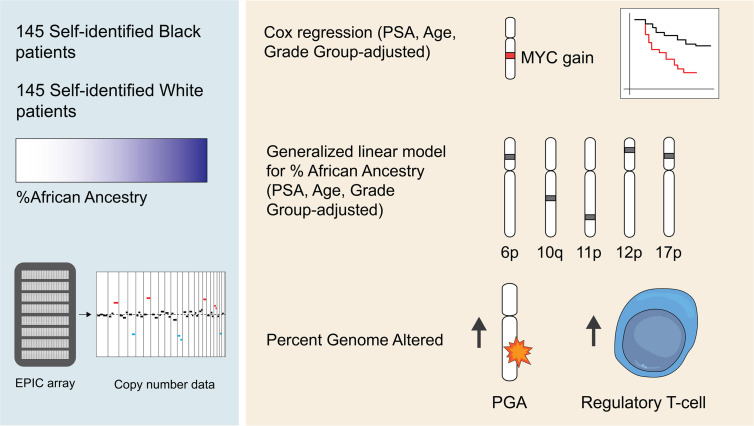

## Introduction

In the United States, self-identified Black (BL) men are more than twice as likely to die from prostate cancer as self-identified White (WH) men (1, 2). While much of this difference is likely attributable to access to care, fewer prostate tumors from BL men have undergone immunogenomic profiling. Intriguingly, published data in relatively small cohorts suggest significant differences in both the genomic (3–16) and immune tumor microenvironment (TME) (4, 9, 17–21) landscapes of prostate tumors from BL compared with WH men, and some of these may be relevant to understanding disparities in outcome between these groups. For example, *ERG* rearrangements (11, 22–24), *PTEN* deletions (7, 11, 23, 24), and *TP53* alterations (14–16, 25) have been identified by our group and others as reproducibly less common among prostate tumors from BL compared with WH men. In parallel, other studies have identified inflammatory gene signatures that are enriched among prostate cancers arising in BL men (4, 9, 17–20, 26).

Though somatic copy number alterations (sCNA) prevail over mutations as the most common genomic alteration in prostate cancer (27, 28) and may be linked to changes in the immune TME (25, 29–32), few prior studies have systematically compared the copy number and immune landscapes of primary prostate cancers using diverse and grade-matched cohorts. Here, we leverage methylation profiling to interrogate the genome-wide copy number landscape and immune cell content of 145 primary prostate tumors from BL men and compare these with a group of tumors from WH men, all surgically treated and with long-term clinical follow-up. BL and WH patients in our cohort were frequency matched by tumor Gleason Grade Group (GG), the widely-used system to pathologically grade primary prostate tumors. To mitigate the effects of genetic heterogeneity among self-identified racial groups, we identified sCNA associated with percent African genetic ancestry. Finally, we are the first to our knowledge to integrate genome-wide copy number profiling with immune tumor content estimation in prostate tumors from diverse cohorts.

## Results

In this retrospective study, we performed Infinium MethylationEPIC profiling on prostate tumors from a previously reported (14, 24, 29) cohort of 290 patients with surgically treated primary prostate cancer and clinical follow-up (Supplemental Figure 1; supplemental material available online with this article; https://doi.org/10.1172/jci.insight.162409DS1). The cohort included tumors from 145 self-identified BL and 145 self-identified WH men, matched for GG at radical prostatectomy, along with 15 normal prostate tissue samples from each group. Clinical and pathologic information for the cohort is summarized in Table 1. Median follow-up for the entire cohort was 6 years, and it was 6 years for BL and 7 years for WH patients. As previously reported for this cohort (33), self-identified race was highly correlated with percent African (Yoruba) ancestry (YRI), with rare exceptions (Supplemental Figure 2).

### EPIC array-derived sCNA are significantly associated with those obtained from targeted sequencing and genomic alterations detected by IHC.

We leveraged the *conumee* package (34) to assess the genome-wide sCNA landscape of tumors from methylation array data. We validated our approach by comparing copy number calls obtained using *conumee* with other methods (e.g., targeted sequencing and Oncoscan SNP arrays) using correlations and correspondence at the top (CAT) plots (35, 36). This latter method allows for assessment of the agreement for genes with large log_2_ fold change, and these genes ultimately are those affected by genomic amplifications or losses. For this analysis, we analyzed 7 cases by EPIC arrays, Oncoscan SNP arrays, and targeted sequencing, which revealed a consistent agreement between copy number calls beyond chance expectations, especially for measurements obtained by EPIC and Oncoscan arrays (Supplemental Figure 3). In our patient cohort, we further tested whether the continuous gene-level sCNA data obtained via *conumee* correlated with previously published sCNA data for 101 genes assessed by targeted sequencing in a subset of our cohort composed of 134 BL patients (14). The correlation between gene-level sCNA data from sequencing and *conumee* was significant and positive for 73 of 101 (72%) genes on the targeted panel (Supplemental Figure 4 and Supplemental Table 1). To further validate the *conumee* sCNA estimations, we examined the continuous *conumee* sCNA data for *PTEN*, *ERG*, and *TP53* stratified by previously published PTEN, ERG, and p53 IHC status (24, 25) using genetically validated protocols for this cohort. As expected, PTEN loss, ERG overexpression, and p53 nuclear accumulation detected by IHC were significantly associated with decreased *PTEN*, *ERG*, and *TP53* copy number (*P* < 0.0001) (Supplemental Figure 5).

### Percent genome altered (PGA) obtained independently through EPIC array and targeted sequencing are correlated.

Next, we performed a segment-level sCNA analysis and derived global sCNA burden estimates expressed as PGA from the EPIC array copy number data. The methylation array-based PGA data were significantly correlated with the previously published PGA estimates obtained from targeted sequencing in a subset of 134 BL patients (Spearman’s rho = 0.44, *P* < 0.0001) (Supplemental Figure 6A). As a final check, we analyzed the associations between PGA and *PTEN*, *ERG*, and *TP53* alterations detected by IHC. Nuclear accumulation of p53 protein is a previously genetically validated surrogate marker of underlying *TP53* missense mutation (37, 38). PGA levels were significantly increased in samples with p53 nuclear accumulation (median = 7.09) compared with those without (median = 3.81) (*P* = 0.02) (Supplemental Figure 6, B–D). We did not observe significant differences in PGA between tumors with intact PTEN (median = 4.53) versus PTEN loss (median = 3.41) (*P* = 0.74). Similarly, tumors with ERG expression indicative of underlying *ERG* fusions did not have increased PGA levels (median = 3.18) compared with ERG^–^ samples (median = 4.65) (*P* = 0.37).

### PGA is not associated with ancestry but is linked to an increased risk of metastasis.

**Figure 1:**
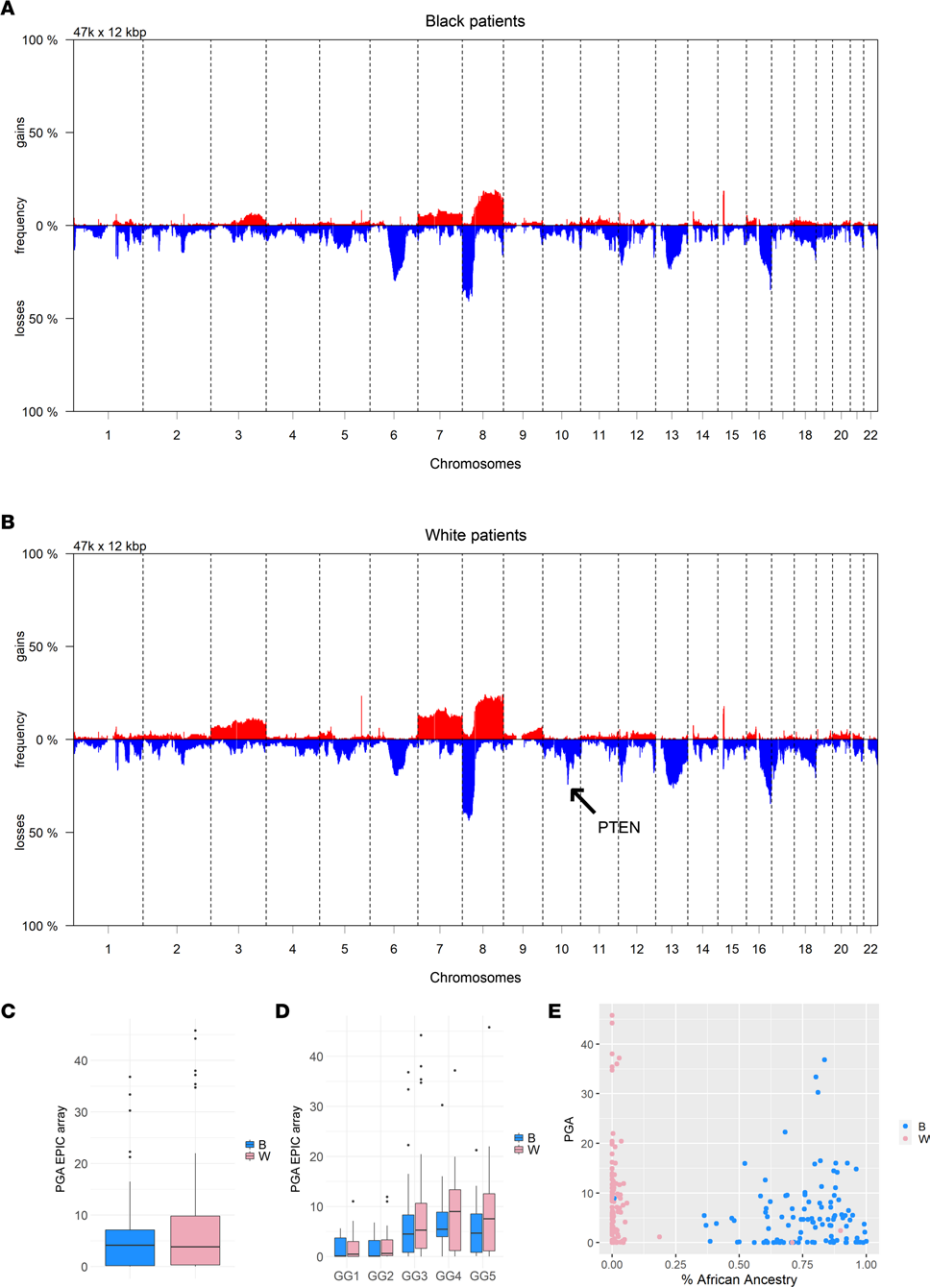
Segment-level somatic copy number alteration (sCNA) landscape of primary prostate tumors for patients who self-identified as Black (BL) or White (WH) . (**A**) Landscape of copy number loss and gain for tumors arising in BL patients (*n* = 145). (**B**) Landscape of copy number loss and gain for tumors arising in WH patients (*n* = 145). The arrow indicates an increased frequency of PTEN loss at 10q23.31 among tumors from WH patients. (**C**) EPIC array–derived percent genome altered (PGA) was not associated with self-reported ancestry (*n* = 249). (**D**) EPIC array–derived percent genome altered (PGA) increases by Gleason Grade Groups, but this relationship was not statistically significant. (**E**) Percent African ancestry (YRI) did not correlate with PGA (*n* = 249).

Having validated the sCNA estimates via methylation array, we next tested associations between PGA and clinical pathologic variables, including self-identified race and genetic ancestry. The segment-level copy number landscape of primary prostate tumors in BL and WH patients highlighted patterns of losses (6q, 8p, 13q, 16q) and gains (3q, chr 7, 8q) that have previously been reported in prostate cancer (9, 27, 28) (Figure 1, A and B). As expected, PGA was significantly associated with GG in the overall cohort by logistic regression analysis as well as separately for each self-identified race group (Supplemental Table 2). Median PGA was 4.13 and 3.82 for BL and WH patients, respectively (*P* = 0.28). Though median PGA was lower among BL compared with WH patients among all GGs, this finding did not reach statistical significance (Figure 1, C and D, and Supplemental Table 2). Similarly, there was no significant correlation between percent African ancestry and PGA in the overall cohort, nor among the self-identified BL or WH patients (Figure 1E).

Median PGA for patients with metastasis was 8.69, while patients without metastasis had a median PGA of 3.49 (*P* = 0.001). PGA was significantly associated with time to metastasis on univariable Cox regression analysis of the combined cohort (*P* = 0.0009) and among self-identified BL (*P* = 0.001) and WH patients (*P* = 0.04) (Supplemental Table 3). In a multivariable Cox regression model adjusted for age, prostate-specific antigen (PSA), and GG, PGA remained significantly associated with metastasis within the entire cohort (*P* = 0.005) and among self-identified BL patients (*P* = 0.002) but not among self-identified WH patients (*P* = 0.11). Similar results were seen when PGA was assessed by quartile in these models (Supplemental Table 3).

### Self-identified race is associated with somatic and germline copy number changes in prostate cancer.

Though self-identified race and genetic ancestry were not significantly associated with PGA, some segment-level differences were apparent in the prostate copy number landscape when compared by self-identified race, including a higher frequency of chr 6 and 10q deletions as well as chr 3 and 7 amplifications, among WH patients (Figure 1, A and B). Thus, we next sought to model gene-level sCNA associated with self-identified race and/or genetic ancestry. Using limma models adjusted for preoperative PSA and GG, we first identified 407 FANTOM-CAT–annotated loci where copy number varied significantly by self-identified race (FDR < 0.05) (Supplemental Figure 7A and Supplemental Table 4). Among common cancer driver genes frequently altered at the copy number level in prostate cancer, only sCNA involving *PTEN* varied significantly by race, with relative increased copy number among the BL compared with WH samples. These data are consistent with a number of previous reports confirming that PTEN gene deletions are less common in prostate tumors arising in BL patients (7, 11, 23, 24), and they validated our models.

However, among the CNAs varying by self-identified race, we noted several loci with previously described ancestry-associated germline copy number variations (gCNV). This was particularly apparent for a cluster of genes lying in a 90 Kb repeat unit on 17q12, including *CCL3L1* (the most potent ligand for CCR5, with 96% homology to CCL3), *CCL4*, and *TBC1D3*, all of which showed significantly increased relative copy number in prostate tumors from self-identified BL patients (Supplemental Figure 7A). This region is well documented to be associated with gCNV, with established higher germline copy numbers — as well as increased gene expression — for *CCL3L1* among African-ancestry individuals compared with European-ancestry individuals (39, 40).

These observations validated that our models were correctly identifying genomic regions with CNVs by race. However, these findings also suggest that the tumor sCNA comparisons could be confounded by underlying gCNV, despite normalization of our sCNA data using a pooled sample of BL and WH normal prostate tissues. In order to systematically identify gCNV associated with self-identified race using the methylation array platform, we leveraged a publicly available normal colon tissue data set, consisting of 80 self-identified BL and 40 self-identified WH subjects with InfiniumEPIC methylation profiling (41). Limma models in the colon data set revealed 1,668 loci (FDR < 0.001) (Supplemental Table 5) with significant gCNV between the 2 self-identified race groups, of which 119 loci overlapped with the sCNA loci discovered in our prostate tumor comparison by self-identified race, comprising 29% (119 of 407) of the identified prostate sCNA (Limma, FDR < 0.05) and prominently featuring the area on 17q12 (Supplemental Figure 7, B and C).

### Increased percent African ancestry is linked to sCNA in prostate cancer.

**Figure 2:**
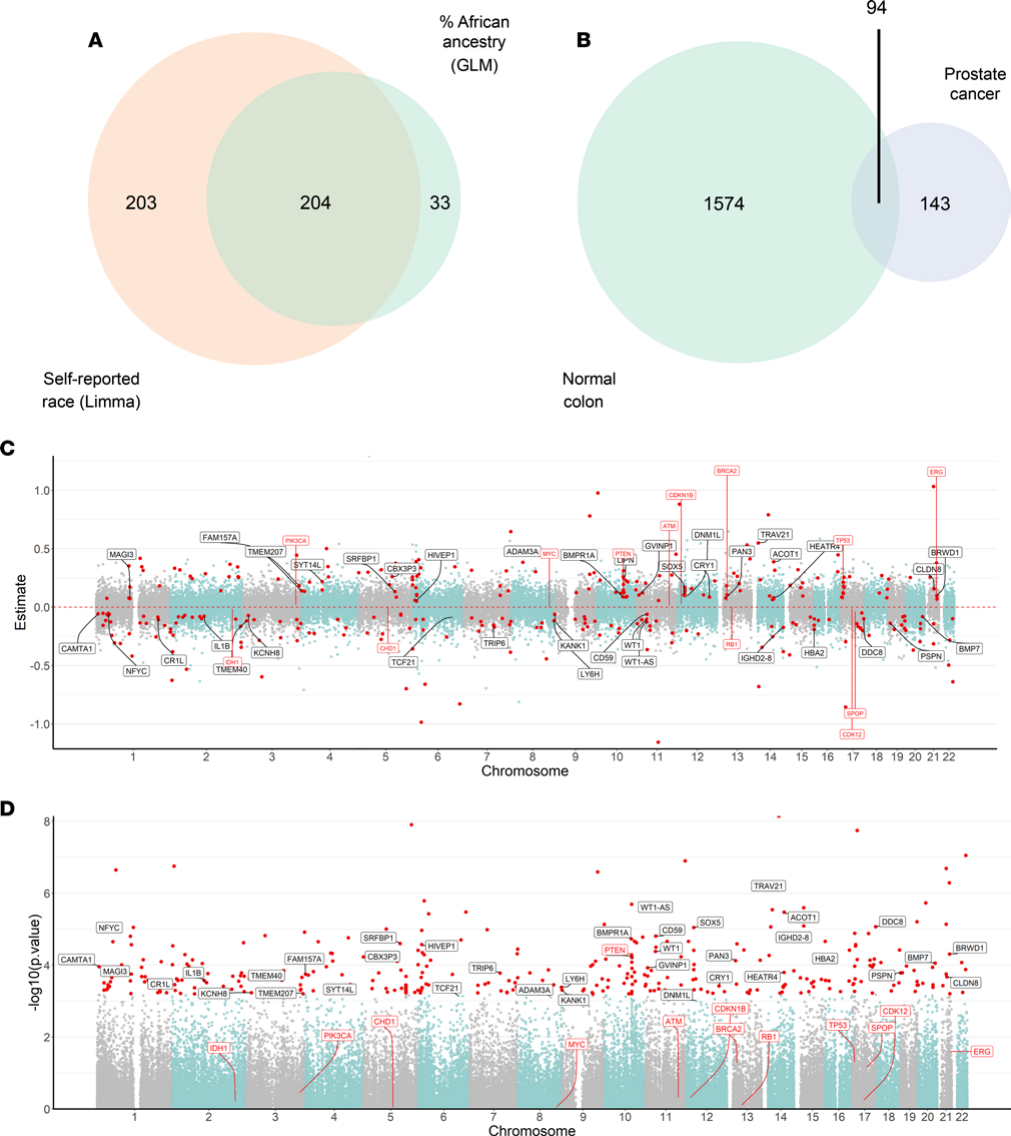
Gene-level sCNA landscape of primary prostate tumors by genetic ancestry. Generalized linear models (GLM) were used to compare the genomic copy number landscape of prostate tumors (*n* = 290) based on percent African ancestry adjusted with Gleason Grade Group (GG1 and GG2 versus GG3, GG4, and GG5), age, and preoperative PSA levels after removing genes likely to represent gCNV based on analysis of benign colon tissue. (**A**) Overlap between loci with sCNA varying significantly by self-reported race or by percent African ancestry (FDR < 0.05). (**B**) Overlap between loci with copy number alteration varying by self-reported race in normal colon tissue (FDR < 0.001) versus prostate cancer by percent African ancestry. From the 237 genes significantly associated with percent African ancestry in the prostate cohort, 94 overlapped with regions found in the comparison between self-identified race in colon normal tissue (Supplemental Table 7). (**C**) Estimates from GLM for each locus shown by chromosome. Each dot represents an individual locus. Red dots show loci with sCNA that vary significantly by genetic ancestry in the GLM using FDR-adjusted *P* < 0.1, with 94 overlapping loci with colon limma models excluded from the figure, as they represent presumptive gCNV. (**D**) GLM *P* values on the *y* axis are distributed by chromosome in the *x* axis. For ease of visualization, labels are shown for a subset of coding loci with adjusted *P* < 0.01, with the complete list available in Supplemental Table 6. Previously described prostate cancer driver genes are indicated with red labels for reference (regardless of significance in model).

Next, we sought to model sCNA associated with genetic ancestry. Generalized linear models (GLMs) adjusted for age, GG, and PSA were performed across the entire FANTOM-CAT–annotated genome to identify how much of the CNAs were explained by percent African ancestry. This comparison yielded 237 loci whose copy number varied significantly by percent African ancestry (FDR < 0.05) (Supplemental Table 6), with 86% (204 of 237) overlapping with the 407 loci found with our limma model conducted with self-identified race (Figure 2A). We then accounted for gCNV present in the GLM results by filtering out loci identified through the colon analysis, which included 94 copy number variable loci likely due to gCNV (Supplemental Table 7) and a total of 143 presumed sCNA that varied by percent African ancestry (Figure 2B and Table 2).

As with the modeling by self-identified race described above, a cluster of genes identified by the GLM on 10q23 (including *PTEN* and *BMPR1A*) showed a higher relative copy number in patients with higher percent African ancestry (Figure 2, B–D, and Supplemental Figure 8). Similarly, a region on 17p near (but not including) *TP53* showed relative higher copy number in patients with increased African ancestry. This is consistent with reports of less common *TP53* mutations in prostate tumors from self-identified BL patients (15), since most *TP53* mutations are accompanied by shallow deletions/loss of heterozygosity (LOH) of the other allele in prostate cancer (28). However, we also identified a number of potentially novel regions with sCNA variable by ancestry, such as a cluster on 11p13 (*WT1*, *WT1-AS*, and *CD59*), with relatively decreased copy number in patients with a higher percentage of African ancestry, and we identified a region centered around 6p24 (including *HIVEP1*) and 12p11-12 (including *SOX5* and *DNM1L*) with increased relative copy number in patients with increased African ancestry (Supplemental Figure 9).

### Chromosome 8q gains are significantly associated with biochemical recurrence and metastasis independent of clinical variables and percent African ancestry.

**Figure 3:**
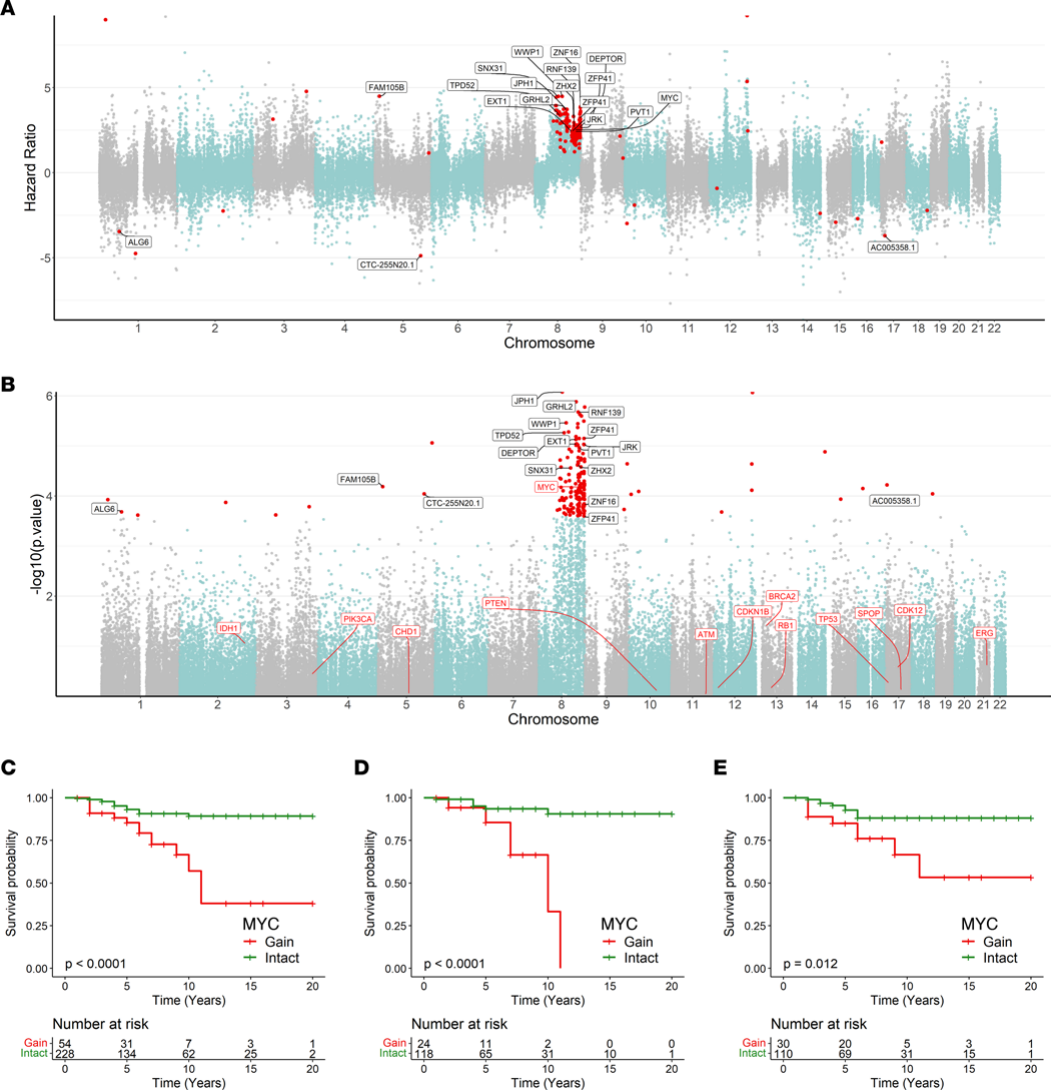
Chromosome 8q gains are significantly associated with risk of prostate cancer metastasis independent of ancestry. Cox regression model adjusted with percent African ancestry, age, preoperative PSA, and tumor Gleason Grade Group (GG1 and GG2 versus GG3, GG4, and GG5) was applied to all samples from our cohort (*n* = 290) and showed significant associations between 8q gains and metastasis. (**A**) Hazard ratio shown by chromosome. Each dot represents an individual locus. Red dots indicate loci where sCNA are significantly associated with risk of metastasis using FDR-adjusted *P* < 0.1). (**B**) *P* values on the *y* axis are distributed by chromosome in the *x* axis. For visualization purposes, only a subset of coding genes are labeled; however, the complete list is available in Supplemental Table 9. Previously described prostate cancer driver genes are indicated with red labels for reference (regardless of significance in model). (**C**–**E**) *MYC* gains (>2 copies) are associated with prostate cancer metastasis in Kaplan-Meier analysis. Log-rank test was employed to identify the association between *MYC* gains and metastasis. (**C**) All patients (*n* = 282). (**D**) BL patients (*n* = 142). (**E**) WH patients (*n* = 140).

Next, we assessed which sCNA were associated with the outcomes of biochemical recurrence and metastasis using age, GG, and PSA Cox regression models adjusted with percent African ancestry. We utilized continuous copy number data across the entire FANTOM-CAT–annotated genome for this analysis. Multivariable Cox regression revealed 162 loci (FDR < 0.1) that were significantly associated with biochemical recurrence independently of percent African ancestry and clinical pathologic variables, including gains in 8q (near but not including *MYC*), losses in 8p, losses in 10q24 (directly adjacent to *PTEN*, including *ATAD1*, which is commonly codeleted with *PTEN*), 12p13 (near *CDKN1B*), and 17p12 (Supplemental Figure 10 and Supplemental Table 8). Cox models for metastasis yielded 175 significant loci (FDR < 0.1). Strikingly, only chromosome 8q gains, including *MYC*, were significantly associated with metastasis independently of percent African ancestry (Figure 3, A and B, and Supplemental Table 9). We confirmed these results via Kaplan-Meier analysis, where *MYC* gains were significantly linked to metastasis for the entire cohort (Figure 3C) and for self-identified BL (Figure 3D) and WH patients (Figure 3E). Overall, *MYC* gains were found in 19% (54 of 282) of samples in our cohort. By self-identified race, 16% (24 of 118) WH and 21% (30 of 110) BL patients had this genomic alteration.

### PGA is linked to increased immunosuppressive TME mediated by Tregs.

**Figure 4:**
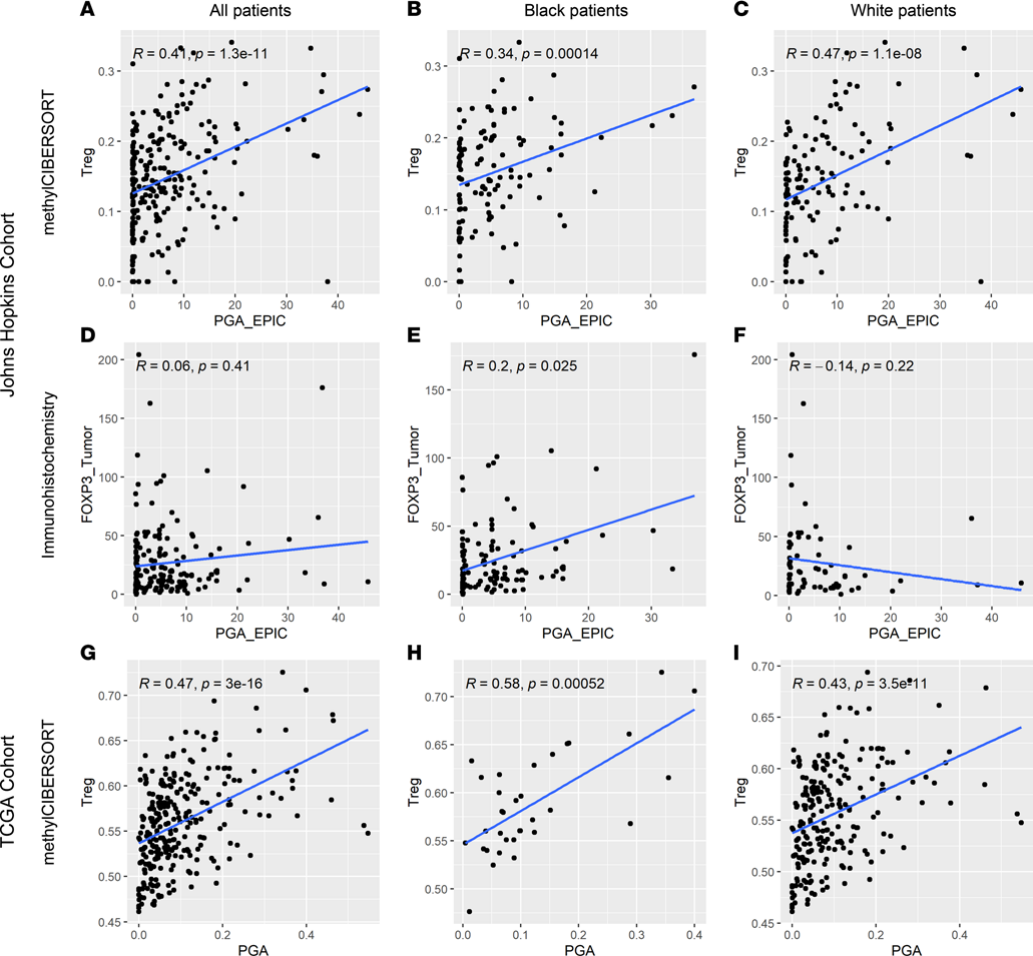
Tregs are significantly associated with PGA independently of self-identified race. (**A**–**C**) Tumor Treg content estimated from methylCIBERSORT was significantly associated with PGA for the entire Johns Hopkins cohort (*n* = 249) (**A**), for BL patients (*n* = 120) (**B**), and for WH patients (*n* = 134) (**C**), independently. (**D** and **F**) FOXP3^+^ cell density quantified by previously reported IHC (29) was not significantly associated with PGA in the entire Johns Hopkins cohort (**D**), nor for WH patients (**F**). (**E**) However, this association was significant for BL patients. (**G**–**I**) Validation using previously published methylCIBERSORT data (42) from the TCGA cohort showed a significant correlation between Treg content and PGA for the entire cohort (*n* = 273) (**G**) and separately in BL (*n* = 33) (**H**) and WH patients (*n* = 222) (**I**).

Since BL and WH patients with prostate cancer have previously been shown to have distinct immune TME profiles (4, 9, 17–21, 26) and since these profiles are associated with tumor-specific genomic alterations (25, 29–31), we next assessed whether PGA was associated with immune cell infiltration and if this association was modified by self-identified race. We leveraged methylation profiling to estimate prostate tumor immune cell content via methylCIBERSORT, as we previously reported for B cells for this cohort (21). Treg immune cell content estimated from methylCIBERSORT was positively and significantly correlated with PGA in the combined cohort and separately for BL and WH patients (Figure 4, A–C). Consistent with this, we found Treg density quantified by a previously published FOXP3 IHC assay (29) was significantly correlated with PGA among BL men, though this correlation was not significant in the overall cohort or among WH men (Figure 4, D–F). We further validated these findings using previously published data from the TCGA cohort (42), where methylCIBERSORT-based estimates of Treg content were positively and significantly correlated with PGA estimates among BL and WH men and overall (Figure 4, G–I). Consistent with the recently reported positive prognostic associations with plasma cell content, and the increased density of these cells in prostate tumors from BL patients (21), B cell content by IHC for CD79a was significantly negatively correlated with PGA in the overall Johns Hopkins cohort (as well as BL and WH subsets) (Supplemental Figure 11). This finding was also replicated among BL patients in the TCGA cohort using methylCIBERSORT, but significant correlations were not found in other comparisons. Immune content estimates for other cellular subsets, including cytotoxic T cells and macrophages, were not consistently significantly correlated with PGA in either cohort (Supplemental Figures 12 and 13).

## Discussion

In prostate cancer, gene deletions, amplifications, and rearrangements prevail over point mutations as the most common genomic alterations, and the total burden of sCNA, expressed as PGA, is the genomic variable most reproducibly associated with GG and clinical outcome in primary disease (43, 44). Though mortality from prostate cancer is more than twice as high among patients who self-identify as BL compared with those who self-identify as WH (1, 2), it is remarkable that few prior studies have systematically compared the copy number and immune landscapes of primary prostate cancers using racially diverse and GG-matched cohorts. In a review of prior studies, the vast majority have included only smaller cohorts (~20 patients in each group), and most larger studies, such as those using TCGA data, have contended with comparing groups that were disparate based on clinical pathologic variables (3–16) (Supplemental Table 10). Compounding these issues, only a few prior studies in unmatched cohorts have integrated genetic ancestry information into these analyses to mitigate against the heterogeneity introduced by social constructs of race and ethnicity (9, 11). Finally, no prior studies, to our knowledge, incorporated long-term clinical follow-up for metastasis and compared sCNA landscape of BL and WH patients with prostate cancer.

Given these limitations, it is unsurprising that few reproducible associations of self-identified race and sCNA have been previously reported. Only 1 major sCNA difference between BL and WH patients has held up across multiple studies using orthogonal technologies: the lower frequency of *PTEN* gene deletions among BL patients. First reported using IHC assays (23, 24), this finding has now been replicated in a number of sequencing studies and is among the few sCNA that have been tested with regard to percent African ancestry (7, 9). The lower rate of *PTEN* deletion among BL patients may be, in large part, due to the established lower frequency of *ERG* gene rearrangement in this population (11, 22–24), since *PTEN* deletion is highly enriched among tumors with *ERG* rearrangement (45, 46) and *ERG* fusions precede PTEN loss in the majority of cases (47, 48). In fact, when patients are stratified by dual PTEN and ERG status, PTEN losses are equally as common among ERG^+^ and ERG^–^ cases, respectively, in BL and WH patients (14) (data not shown). It is curious that the frequency of a genomic alteration with an established connection to poor outcomes, such as *PTEN* deletion, is actually lower among BL compared with WH men, despite the increased mortality in this population. Given that *PTEN* deletion has been associated with increased genomic instability in prostate cancer (49), this finding raises the question of whether PGA might be lower among prostate tumors from BL compared with WH men, and if not, whether there are other genomic alterations driving adverse outcomes that may be more common among BL men.

To begin to answer these questions, in the current study, we combined in silico and tissue analyses for a grade-matched cohort of nearly 300 primary tumors from self-identified BL and WH men with accompanying genetic ancestry data, to model the association of sCNA with self-identified race and clinical outcomes. Though sCNA are typically queried using SNP arrays or array comparative genomic hybridization (aCGH), methylation array data can be used with similar sensitivity (50, 51). We leveraged InfiniumEPIC data via the *conumee* platform to infer copy number across more than 70,000 coding and noncoding loci using the extended FANTOM-CAT genome annotations. Because we are not aware of previous studies taking a similar approach for prostate cancer, we extensively compared our gene-level sCNA estimates to panel-based sequencing and Oncoscan data for a subset of BL patients, as well as to genetically validated and previously published PTEN and p53 IHC assays in the entire cohort (25, 29). In addition, we compared methylation-based PGA to previously published PGA data based on targeted sequencing of 101 genes in a subset of BL patients (14), though this comparison was somewhat limited in that it only included a subset of patients and a panel of 101 genes.

As a final clinical validation of our methodology, we demonstrated that the methylation-based PGA estimates were associated with GG (with higher grade tumors showing increased PGA) as well as with risk of metastasis in models adjusted for clinical pathologic variables. Notably, we did not see significant differences in PGA by either self-identified race or percent African ancestry. Though tumors from BL patients had lower absolute median PGA across GGs, these differences were not statistically significant. This suggests that, while frequencies of specific alterations such as *PTEN* deletion may vary by ancestry, PGA remains similar for primary tumors between BL and WH men and may not contribute to observed differences in clinical outcomes based on our single-institution cohort composed of African-American patients. Additional studies with more diverse populations are required to validate our findings.

We next set out to identify gene-level sCNA differences between BL and WH patients. Using linear models to compare the 2 groups while adjusting for clinical pathologic variables, we identified 407 genomic loci where copy number differed by self-identified race. Validating this methodology, PTEN was identified as one of the only known prostate cancer drivers on this list, consistent with previous studies (7, 11, 23, 24). Given that we had normalized our tumor copy number data using a reference cohort of benign tissues from BL and WH men, we were surprised to also find among the 407 loci several other genes where germline copy number has been reported to vary by genetic ancestry. Many of the genes with the highest effect sizes in our model mapped to segmental duplications, including a region on chromosome 17q12 containing *CCL3L1*, as well as *CCL3* and *CCL4* (52). These genes also appeared in models using percent African ancestry as the output, which were generally highly overlapping with models using self-identified race. *CCL3L1* is the paralogue of *CCL3*, encoding the most potent ligand for CCR5, which also serves as a coreceptor for HIV (39). Because of this association, gCNV of the *CCL3L1* locus have been investigated in depth, especially with respect to gene expression of *CCL3L1* in T cells. Both genomic copy number and transcript levels for *CCL3L1* are significantly higher in patients of African compared with European ancestry, with median germline copy numbers of 4 (range 1–11) and 2 (range 0–9), respectively, and a linear relationship between genomic copy number and T cell gene expression (39). Consistent with this, we found that *CCL3L1* was overexpressed in BL prostate cancer tumors from the TCGA cohort compared with WH patients (Supplemental Figure 14). Nearby, *CCL4L1* and *TBC1D3* also exhibit gCNV, with higher copy numbers in African patients (40). While *CCL3* and *CCL4* are reported to be germline copy number invariant by quantitative PCR (qPCR) assays (53), they often appear as copy number variable in array-based genome-wide studies (including this study) (40), likely due to the high level of homology with their respective isoforms, and this igh level of homology makes them difficult to distinguish in many assays due to nonspecific hybridization.

While the finding of higher copy numbers of *CCL3L1*, *CCL4*, and *TBC1D3* validated our models comparing patients by self-identified race and genetic ancestry, these data suggest that our sCNA list also inadvertently contained gCNV. To more systematically exclude contributions from gCNV in our tumor CNA comparison, we leveraged a public EPIC methylation data set derived from benign colon tissue from both BL and WH patients. Comparing copy number stratified by self-identified race using limma models in this data set, we comprehensively identified more than 1,600 loci that are likely to be affected by gCNV, which comprised nearly 30% (119 of 407) of the loci identified in our prostate tumor comparison by self-identified race and 40% (94 of 237) of the loci identified in our prostate tumor comparison by genetic ancestry. To our knowledge, we are the first group to leverage methylation profiling of benign tissues to discern gCNV by self-identified race.

In retrospect, the confounding effect of gCNV is apparent in several previous prostate cancer studies that have compared BL and WH patients. For example, a prior aCGH study comparing 20 BL and 21 WH prostate tumors found a region within 17q12 to be an area of relative gains among BL patients, though a germline contribution was not acknowledged (4). Strikingly, *CCL3* and *CCL4* also comprise 2 of the immune genes with reproducibly increased expression in BL compared with WH prostate tumors, a finding replicated in multiple independent array-based studies (20, 26, 54) but conspicuously absent from RNA-Seq–based studies such as TCGA (Supplemental Figure 14). Given the high level of homology of these isoforms to *CCL3L1* and *CCL4L1*, where copy number and prostate gene expression are established to be higher in individuals of African descent (Supplemental Figure 14), it seems likely that these findings are interrelated and a consequence of previously unrecognized germline copy number differences. Future studies will be required to determine whether *CCL3L1* and *CCL4L1* expression levels may be misclassified as *CCL3* or *CCL4*, respectively, in published gene expression data sets.

After excluding gCNV, we identified close to 150 loci where sCNA varies by percent African ancestry, a list that highly overlapped with comparisons by self-identified race. In addition to the region around *PTEN*, which showed higher relative copy number in patients with higher African ancestry, similar findings were noted for a region on 17p near *TP53*, consistent with reports of more infrequent *TP53* alterations in prostate tumors from self-identified BL patients (14–16, 25). Notably, we also identified a number of potentially novel regions with CNVs by ancestry, including clusters on 11p13, 6p24.1, and 12p12.1 (Table 2). These regions have not been identified as differing by ancestry in previous studies (Supplemental Table 10). However, 11p13 has previously been reported to contain a putative metastasis suppressor for prostate cancer (55), and 12p12 has been reported to be affected by deletions in predominantly WH cohorts (56). Future studies may delve into the functional and clinical effects of deletions in these regions that appear to be less common among BL patients. Of interest, the 8q region (including *MYC*), which was most closely associated with risk of metastatic disease in our cohorts, did not show significant CNV by genetic ancestry in our analyses, despite prior reports suggesting more frequent amplification in BL men in clinically unmatched cohorts (9, 16). Due to the underrepresentation of BL patients in most genomic studies of prostate cancer, additional large, matched cohorts will be useful to confirm our findings.

Previous studies have demonstrated distinct patterns of immune cell infiltration in the TME from self-identified BL and WH patients with prostate cancer (4, 9, 17–21). In parallel, in silico investigations have demonstrated that increased levels of aneuploidy — a measure of chromosomal instability — are linked to lower immune response in several tumor types (30, 57), though prostate cancer (a classically immunologically “cold tumor”) did not show a strong association in these studies. Herein, we leveraged tumor immune content estimates via methylCIBERSORT and assessed whether they were correlated with PGA in a diverse cohort. Strikingly, we found that PGA, a measure of the percent of the genome affected by genomic losses or gains, was positively correlated with increased Treg content in tumor tissue, an observation that held true for both self-identified races and one that was validated using previously published IHC for FOXP3^+^ cell densities in BL patients (29) and methylCIBERSORT data from the TCGA cohort (42). This finding is reminiscent of a recent observation from our group that Treg content by methylCIBERSORT in the TCGA cohort is increased in tumors with higher homologous recombination deficiency (HRD) score (42), another measure of genomic instability that integrates whole-genome tumor LOH, telomeric allelic imbalance, and large-scale state transition scores (58). Notably, we found the association of Treg content with PGA was significantly stronger than the association of FOXP3^+^ cell density with PGA. This might be due to the nearly 10-fold larger volume of tissue sampled for DNA isolation compared with tissue microarray preparation, and this nearly 10-fold larger volume of tissue may better capture intratumoral heterogeneity in immune cell content.

Consistent with our previous observation of improved prognosis with increased B cell content in primary prostate cancer (21), we found that B cell densities by IHC were inversely correlated with PGA, which is itself an adverse prognostic marker. This finding held up in both self-identified race groups in the Johns Hopkins cohort but was only replicated among BL patients in the TCGA cohort via methylCIBERSORT, perhaps due to higher B cell content in these tumors, as we have previously described (21). Notably, however, we were unable to replicate this in the methylCIBERSORT data from the Hopkins cohort. Taken together, these findings suggest that immunosuppressive T cell content is positively correlated with measures of genome-wide genomic instability regardless of race in primary prostate cancer, while there is a weaker negative correlation for B cells and PGA. Future studies will be required to parse out the extent to which these findings are causal or confounded by associations with other variables.

Our study has several limitations. First, we were unable to obtain germline blood or saliva samples from the patients due to the long interval since treatment in this cohort. The lack of these data precluded germline normalization on a case-by-case basis, and we instead used a pooled subset of normal prostate tissues for normalization and a large race-matched cohort of normal colon tissue samples for filtering for gCNV. Though we do not have evidence of tissue-specific gCNV that might affect colon and not prostate, we cannot completely exclude this possibility. Second, though we conducted several checks to validate our EPIC-derived sCNA data — including comparison with SNP-based sCNA assessment, in some cases — and found good agreement, it is possible that sCNA assessment via methylation profiling systematically over- or undercalls sCNAs at some loci. Third, to account for potential losses and gains occurring randomly, we increased the density of our investigated loci by employing FANTOM-CAT–annotated genomes, which contain > 70,000 coding and noncoding loci. However, many regions with lower density of loci may remain undetected, and structural rearrangements, such as translocations, were not assessed. Lastly, this is a single-institution and single-region cohort study. Therefore, additional studies with patients from other institutions and other geographic regions are necessary to validate these findings.

### Conclusions.

Herein, we have leveraged methylation profiling to define the sCNA and immune landscape of primary prostate cancer in a diverse cohort with integrated genetic ancestry information and clinical follow-up. After extensive validation, we provide the first comprehensive mapping to our knowledge of genomic loci where loss or gain is associated with genetic ancestry, and we demonstrate that the global sCNA burden of prostate tumors does not vary by ancestry. We note that gCNV are potential confounders in sCNA analyses and may account for differences in immune gene expression previously reported by other groups for prostate tumors arising in BL versus WH patients. Finally, we identify an association between global burden of sCNA and Treg infiltrate that is independent of ancestry.

## Methods

### Samples.

Our study cohort has been previously described (14, 24, 29) and comprised 290 patients with prostate cancer treated with radical prostatectomy at the Johns Hopkins Hospital or Bayview Medical Center (Baltimore, Maryland, USA), including 145 men self-identified as BL and 145 men self-identified as WH. Radical prostatectomies were performed between 1995 and 2010. For GG 1 and 2, which were relatively more common, 50 BL men with clinical follow-up were randomly selected from the total number of consecutive cases during that time period. For other GG categories, all BL men meeting criteria were included. Within each GG, an equivalent number of prostatectomy samples from self-identified WH men were selected from the same time period via stratified random sampling to provide a grade-matched WH cohort for comparison. Of this final cohort of 391 cases, we selected 290 grade-matched samples with at least 500 ng of tumor DNA required for methylation analyses. The entire 290-sample cohort was used for further analyses. However, methods employing quality control thresholds and other cutoffs led to a reduced sample size for a few comparisons (Supplemental Figure 15).

Normal adjacent prostate tissue was obtained from a subset of 30 randomly selected patients from the above cohort with adequate benign tissue in the radical prostatectomy specimen, including 15 samples from BL and 15 samples from WH men (Supplemental Table 11). In the vast majority of cases (28 of 30), a completely normal radical prostatectomy quadrant section, without any tumor present, was used to sample benign prostate for normal DNA isolation. These blocks were typically several centimeters from tumor. In cases where most blocks were extensively involved by tumor, we sampled adjacent normal tissue from tumor-containing blocks, as long as it was > 3 mm from the tumor sample. Genetic ancestry estimation on this cohort was obtained through SNP array data (Global Screening Array v3, GSAv3), as previously published (33).

### Infinium MethylationEPIC BeadChip arrays.

Tumor regions with at least 50% tumor cellularity by pathologic examination were visually annotated for the dominant tumor nodule (highest GG), and 4–6 tumor punches (0.6 mm diameter) were obtained in these regions for DNA isolation. In a prior publication utilizing tumor DNA samples from BL patients in this cohort (14), the median variant allele frequency for SPOP mutations (23%) and FOXA1 (27%) suggested that our visual estimates of 50% tumor cellularity are reasonable; however, visual estimation may be inaccurate. DNA from formalin-fixed paraffin-embedded tumor regions was obtained as previously described (14). All tumors and benign samples were profiled via Infinium MethylationEPIC BeadChip (Illumina), which covers 850,000 methylation sites at single-nucleotide resolution. IDAT files containing red and green channels from the methylation output were combined for each sample using the *minfi* package in R (59). Quality control checks performed via *shinyMethyl* (60) were used to identify abnormally distributed probe quantification sites. Normalized data were obtained through the Subset quantile Within-Array Normalization (SWAN), which normalizes Infinium type I and II probes simultaneously. We confirmed that our experiments and analyses were in line with previous findings confirming the hypermethylation state of *GSTP1* and *PTGS1* in tumor compared with normal samples (data not shown). Raw and processed methylation data are available on the Gene Expression Omnibus (GEO) repository (GSE221219; https://www.ncbi.nlm.nih.gov/geo/query/acc.cgi?acc=GSE221219).

### sCNA assessment from EPIC array methylation assays using conumee.

We used *Methylset* objects obtained from *preprocessRaw* functions in *minfi* to derive whole-genome copy number data using the *conumee* package in R (34), which has been used for CNA estimation in many tumor types (61–67). The 30 normal samples were used as the reference genome to calibrate the copy number calls for the tumor cohort. Segmented regions of copy number change derived from *conumee* were then used in further analyses. For gene-level copy number estimation, we leveraged a comprehensive genomic annotation using the permissive set of the FANTOM-CAT (68, 69), which is composed of 124,245 coding and noncoding gene annotations. These loci were then overlapped with those from EPIC array, which yielded 70,198 loci. This annotation increased the density of genes and other regions in the genome to improve our ability to determine copy number regions more precisely. To visualize and compute region-level frequency of genomic gains and losses, we used the *CGHcall* package (70) to obtain regions that were altered by self-identified race.

### Identification of sCNA for self-identified race in prostate cancer.

Aiming to identify copy number loci that varied according to self-identified race, we employed a GLM approach (*limma*) (71) using the continuous gene-level copy number data derived from our EPIC array methylation analysis. These models were adjusted with preoperative PSA, age, and GG (with GG1 and GG2 grouped as low-risk and GG3, GG4, and GG5 grouped as high-risk). *P* values were adjusted using the Benjamini-Hochberg method.

### Assessment of gCNV by self-identified race.

To filter out CNAs varying by race that might be due to underlying gCNV, we leveraged a large publicly available EPIC array methylation data set encompassing 128 normal colon samples, of which 88 were from BL and 40 were from WH healthy individuals without a history of any cancer diagnosis or a family history of colon cancer syndromes (GSE151732) (41). The colon data were processed, normalized, and analyzed via the same bioinformatic pipeline used for our data, comparing cases by self-identified race in order to identify gCNV. Genomic regions/genes of copy number change between BL and WH individuals identified in the normal colon samples were deemed to be germline and removed from all subsequent analyses when overlapping those identified in prostate cancer.

### Modeling sCNA associated with percent African ancestry.

To complement our analyses and findings based on self-identified race, we used a GLM approach. To this end, we used the EPIC-derived continuous copy number data to model the percent African ancestry in our prostate cancer cohort, adjusting by GG, age, and preoperative PSA levels. *P* values were adjusted using the Benjamini-Hochberg method.

### Modeling biochemical recurrence and metastasis.

Next, we performed multivariable Cox proportional hazards regression to identify loci (genes and lncRNAs) associated with biochemical recurrence and metastasis in our cohort. These models were also adjusted for GG, PSA, age, and percent African ancestry. The Benjamini-Hochberg method was used to adjust for multiple comparisons. Gene-level copy number gains and losses obtained from the *CGHcall* package were used to validate our findings from Cox regression models.

### Segment-level and PGA of sCNA calls.

We next obtained PGA estimates for each sample from EPIC array copy number data obtained from *conumee* at the genomic segment level. We used the expectation-maximization (EM) algorithm to model the copy number distribution in the complete prostate cancer cohort as a mixture of 3 normal distributions — representing gained, lost, and intact copy number regions; we then assigned each segment for each case to 1 of such normal distributions. In order to calculate the PGA, we obtained the genomic coordinates for all segments, calculated the cumulative size of those deemed gained or lost separately for each sample, and then divided such sums by the whole genome size. In this analysis, 36 samples did not have any segmental sCNA that passed our cutoff points and were not used in downstream analyses. We validated the PGA data obtained from the EPIC array platform with sequencing-derived PGA data from a subset of 134 patients from our cohort. PGA was also used in logistic regression and Cox regression models adjusted by GG, PSA levels, and age.

### Estimation of tumor immune cell content.

To identify the association between PGA and immune cell content, we used *methylCIBERSORT* (72), a methylation-based deconvolution method to obtain cell density scores for CD14 cells (macrophages), CD19 cells (B cells), CD4 effector T cells, CD56 cells (NK cells), CD8 T cells, endothelial cells, eosinophils, fibroblasts, neutrophils, and Tregs, as previously published for B cells in this cohort (21) and in the TCGA cohort (42). We used previously published IHC data from our cohort to validate our in silico deconvolution method. Markers for Tregs (FOXP3), B cells (CD79), CD8 T cells, and macrophages (CD163) were employed as described previously (21, 29, 33).

### Availability of data and materials.

InfiniumEpic Methylation array data are pending deposition in GEO. Public-domain data set used (https://www.ncbi.nlm.nih.gov/geo/query/acc.cgi?acc=GSE151732).

### Statistics.

Fisher’s exact and χ^2^ tests were used to compare self-identified race groups for categorical data. Mann-Whitney *U* test was used for clinical continuous data. Linear regression models were conducted to determine sCNA status by genetic ancestry. Spearman’s correlation was used to determine the relationship between immune cells and PGA. *P* value threshold was set to 0.05, and Benjamini-Hochberg FDR was set to 0.1 or less as described for each analysis.

### Study approval.

This study was approved by the Johns Hopkins IRB in accordance with the US Common Rule under a waiver of consent to enable research on excess archival biospecimens (IRB00208526).

## Author contributions

TLL, TV, and LM conceived the study. FF, SM, HK, SZ, JX, KSS, ELI, AAM, WBI, and EMS contributed to data collection and analysis. TLL, TV, and LM drafted the manuscript. All authors critically reviewed the manuscript and agreed to submit for publication.

## Supplementary Material

Supplemental data

Supplemental tables 1-22

## Figures and Tables

**Table 1:**
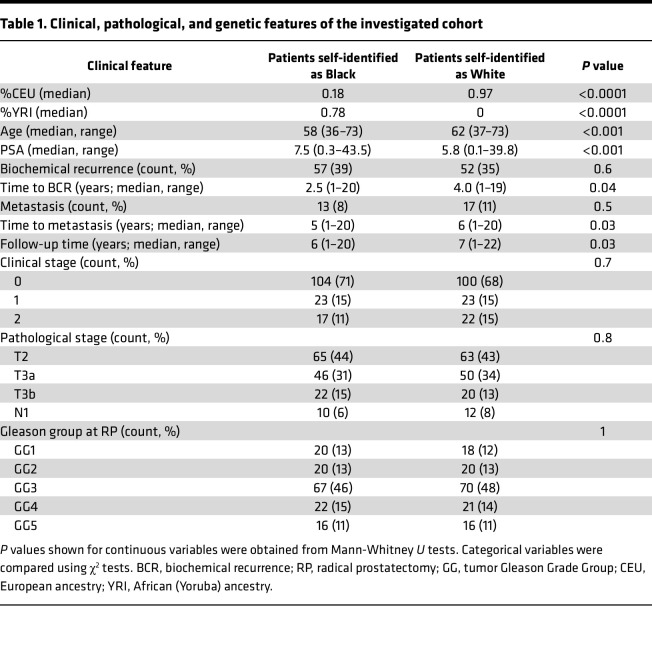
Clinical, pathological, and genetic features of the investigated cohort

**Table 2:**
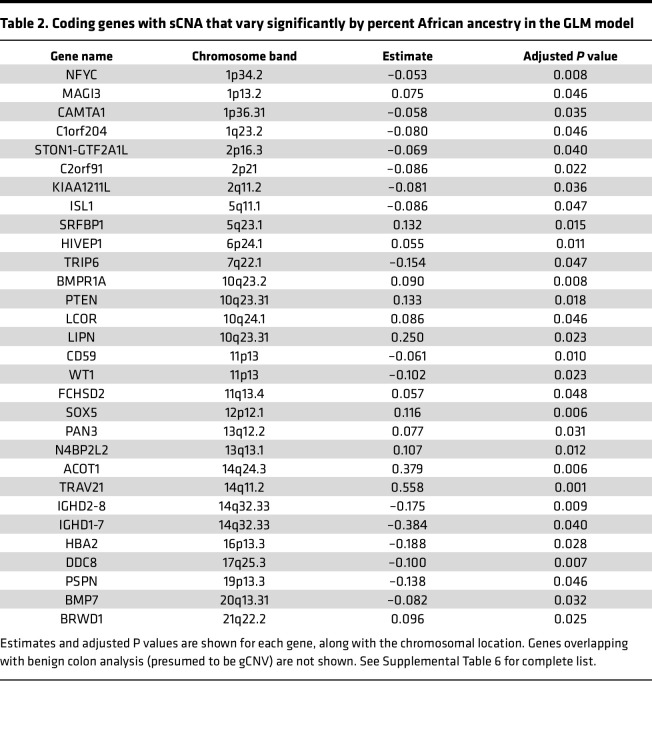
Coding genes with sCNA that vary significantly by percent African ancestry in the GLM model

## Version 1 02/08/2023

Electronic publication
