# Supplementary material for: Association of self-identified race and genetic ancestry with the immunogenomic landscape of primary prostate cancer
Source: JCI Insight. 2023 Feb 8;8(3):e162409. doi: 10.1172/jci.insight.162409 (PMC9977441; doi:10.1172/jci.insight.162409)

Supplementary Figure S1 - Flowchart of study design. EPIC-array data were used to derive copy number and immune-cell abundance for 290 prostate tumor samples. Segment and gene-level input data were used to obtain PGA and to perform multivariable models, respectively. JHMI – Johns Hopkins Medical Institutions, EM – Expectation maximization; GLM – generalized linear model; PGA – percent genome altered, sCNA – somatic copy number alteration, %A – percent African ancestry, GG – Gleason Group, PSA – preoperative prostate-specific antigen, Tregs – regulatory T-cells.

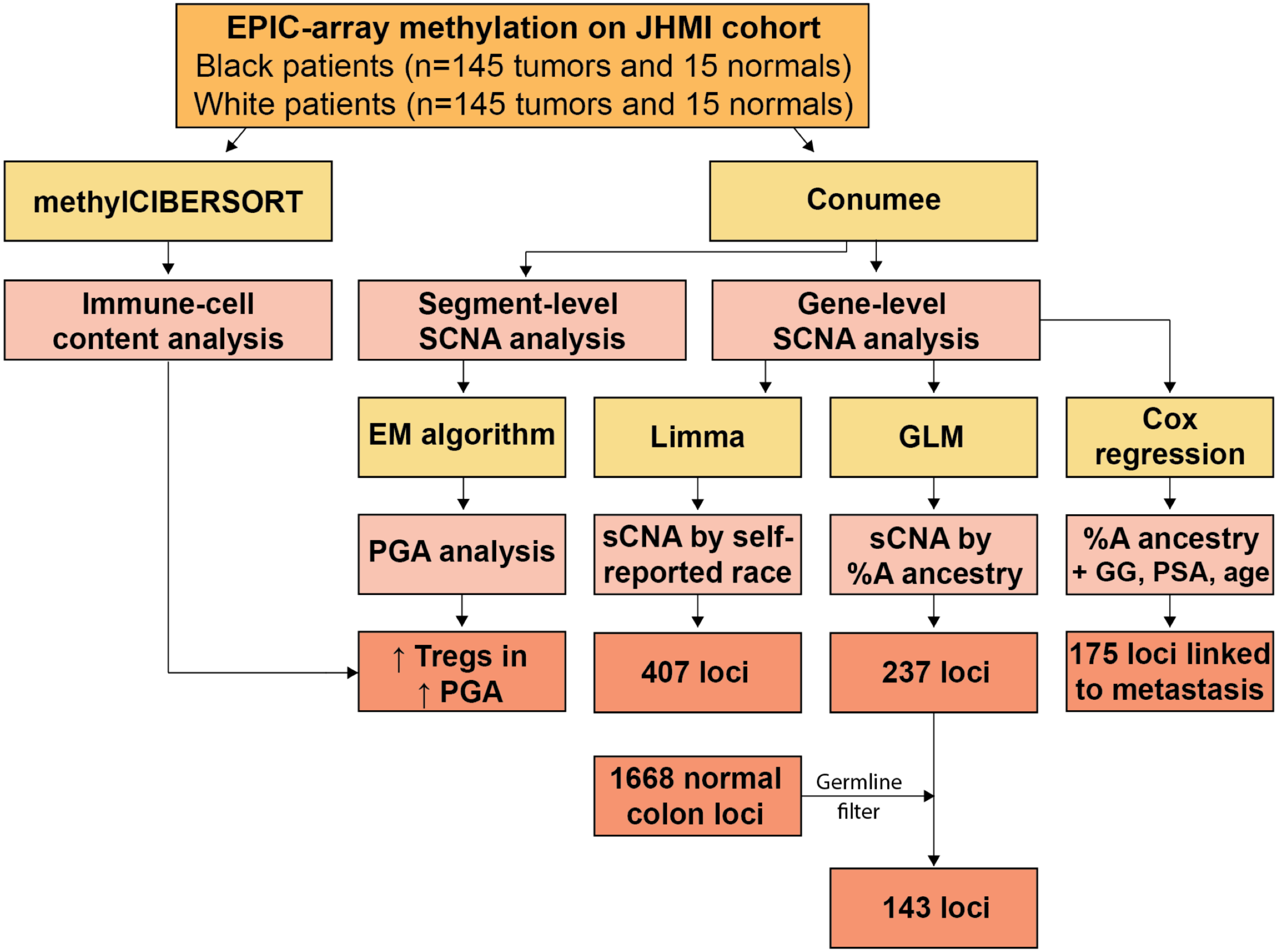

Supplementary Figure S2 – Frequency of BL (n=145) and WH (n=145) patients by percent African (YRI) via previously published SNP array analysis (33).

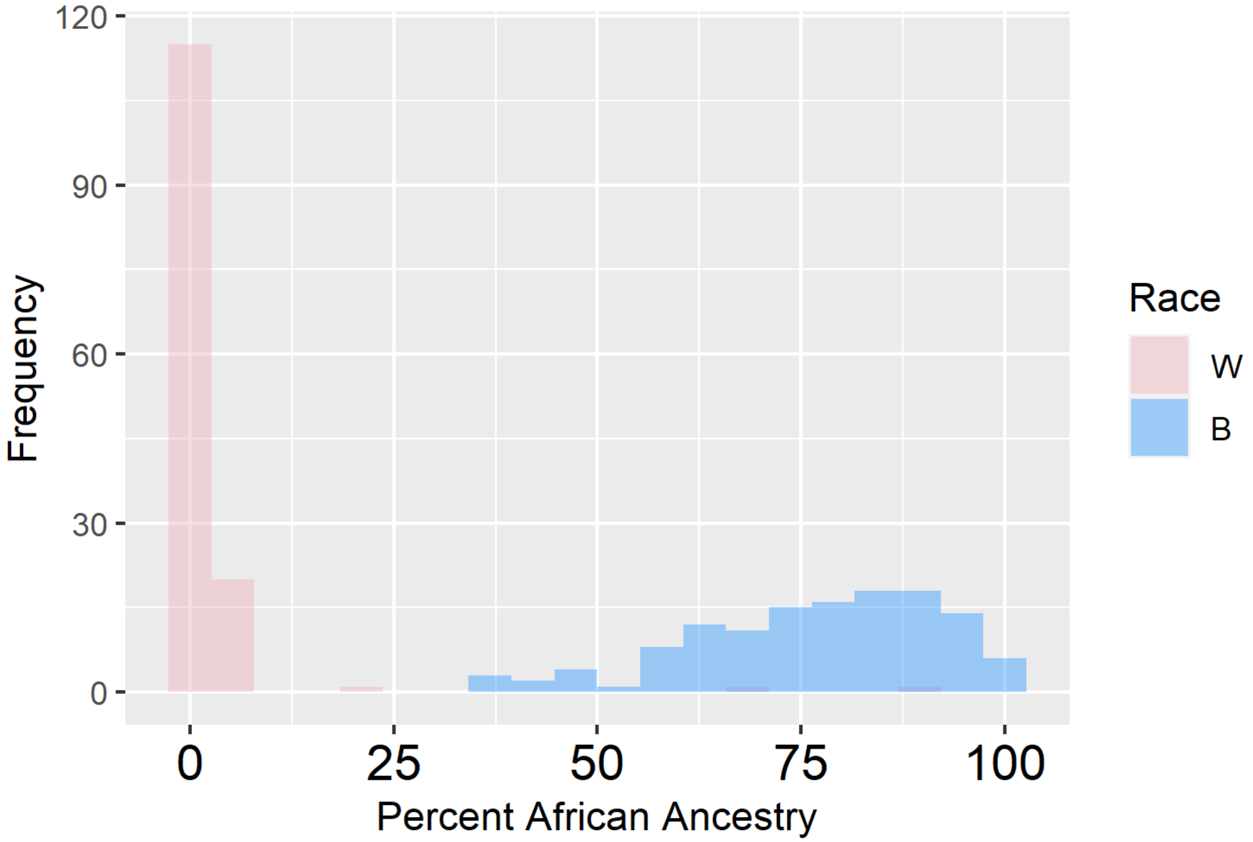

Supplementary Figure S3 – Correlation plots and CAT plots for a subset of 7 samples showing high concordance between sequencing, EPIC-array SCNA, and Oncoscan data. (Continues)

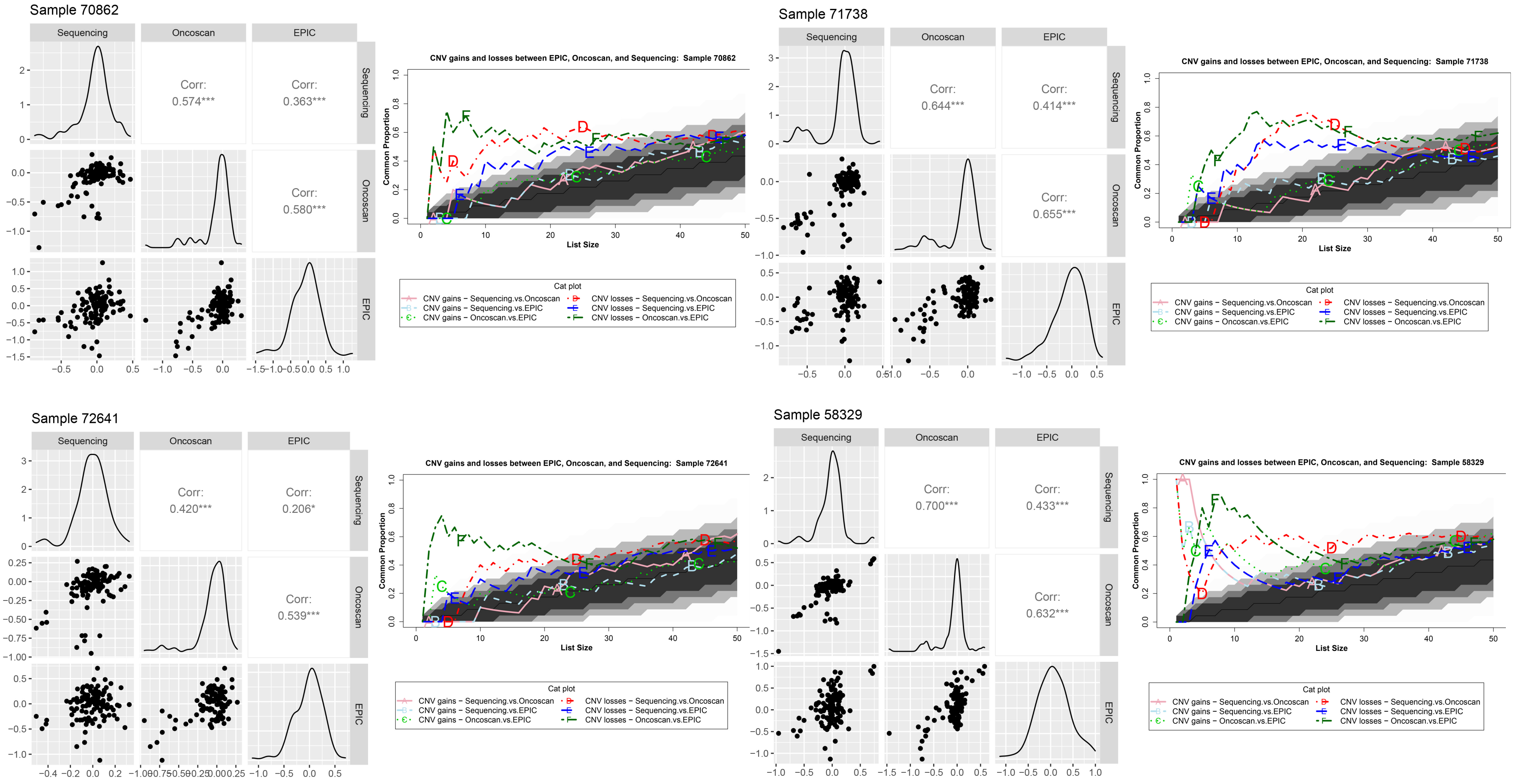

Supplementary Figure S3 – Correlation plots and CAT plots for a subset of 7 samples showing high concordance between sequencing, EPIC-array SCNA, and Oncoscan data.

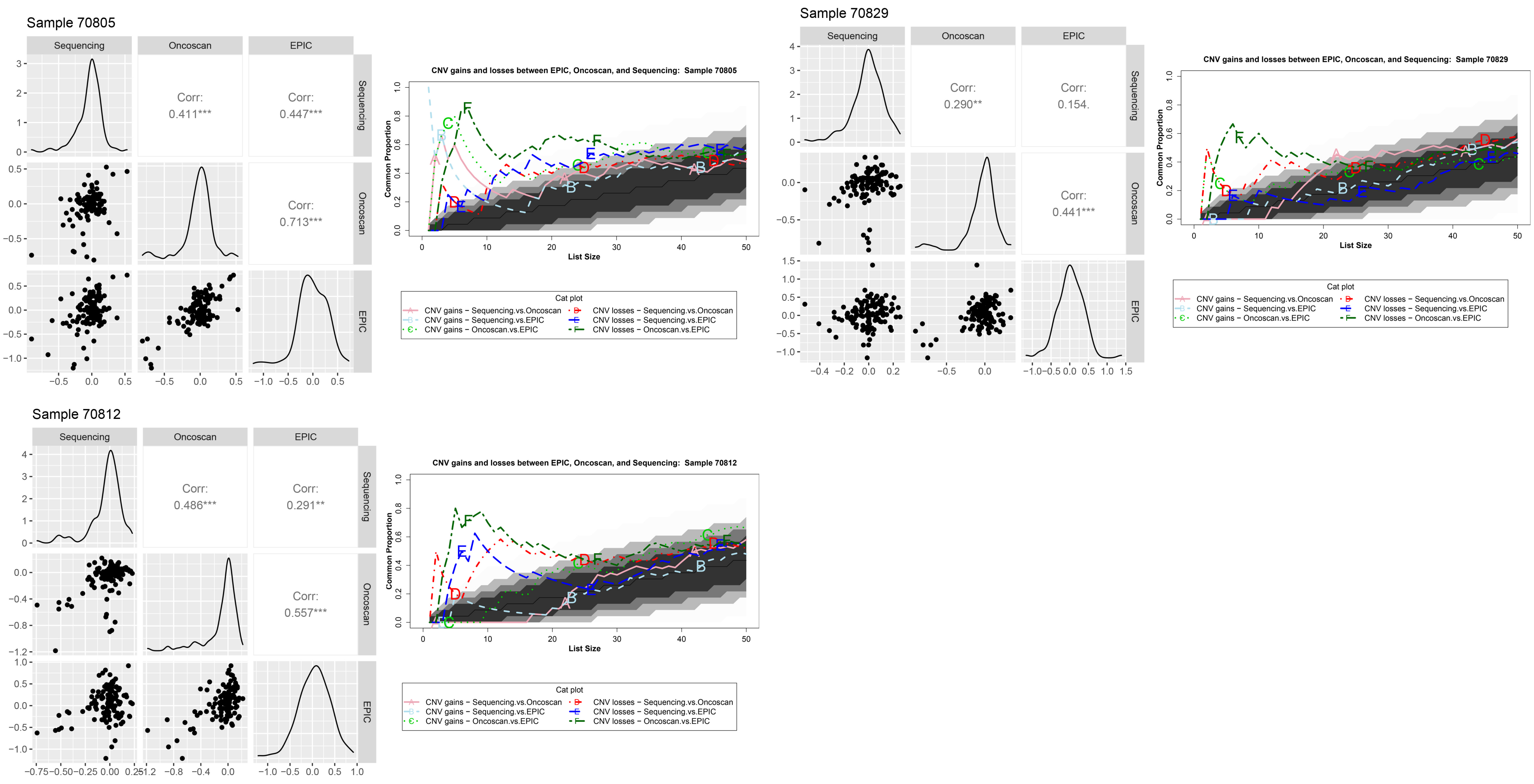

Supplementary Figure S4 – EPIC array copy number data (EPIC) for 101 genes correlated with copy number estimates from previously published panel-based sequencing (seq) for a subset of BL patients (n=134) in our cohort.

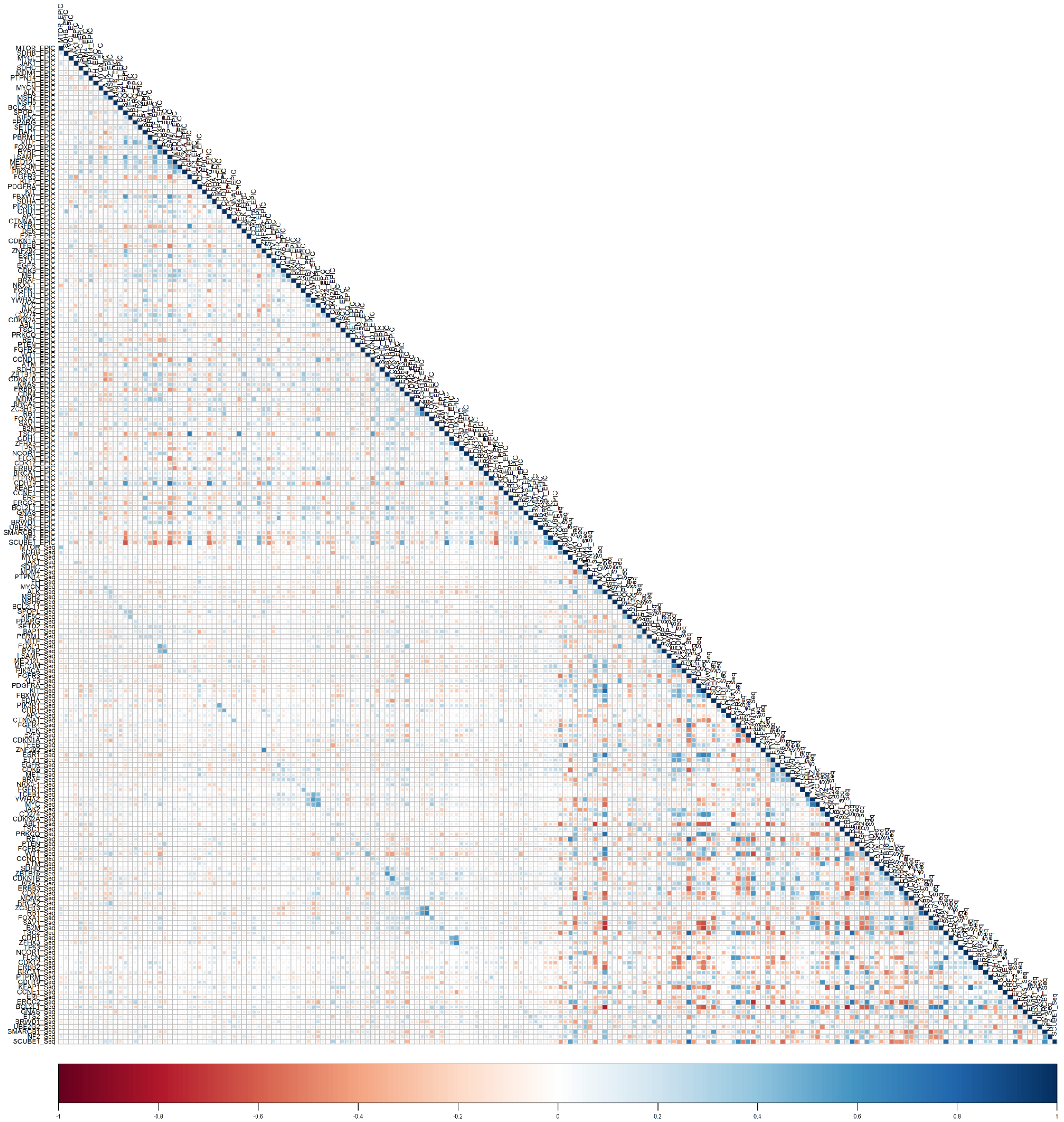

Supplementary Figure S5 – EPIC-derived sCNA data for prostate cancer drivers. (A) PTEN loss by immunohistochemistry was significantly associated with lower *PTEN* copy number by EPIC array. Similarly, ERG positivity (B) and p53 nuclear accumulation (C) by immunohistochemistry were significantly associated with lower *ERG* and *TP53* EPIC-derived copy numbers, respectively.

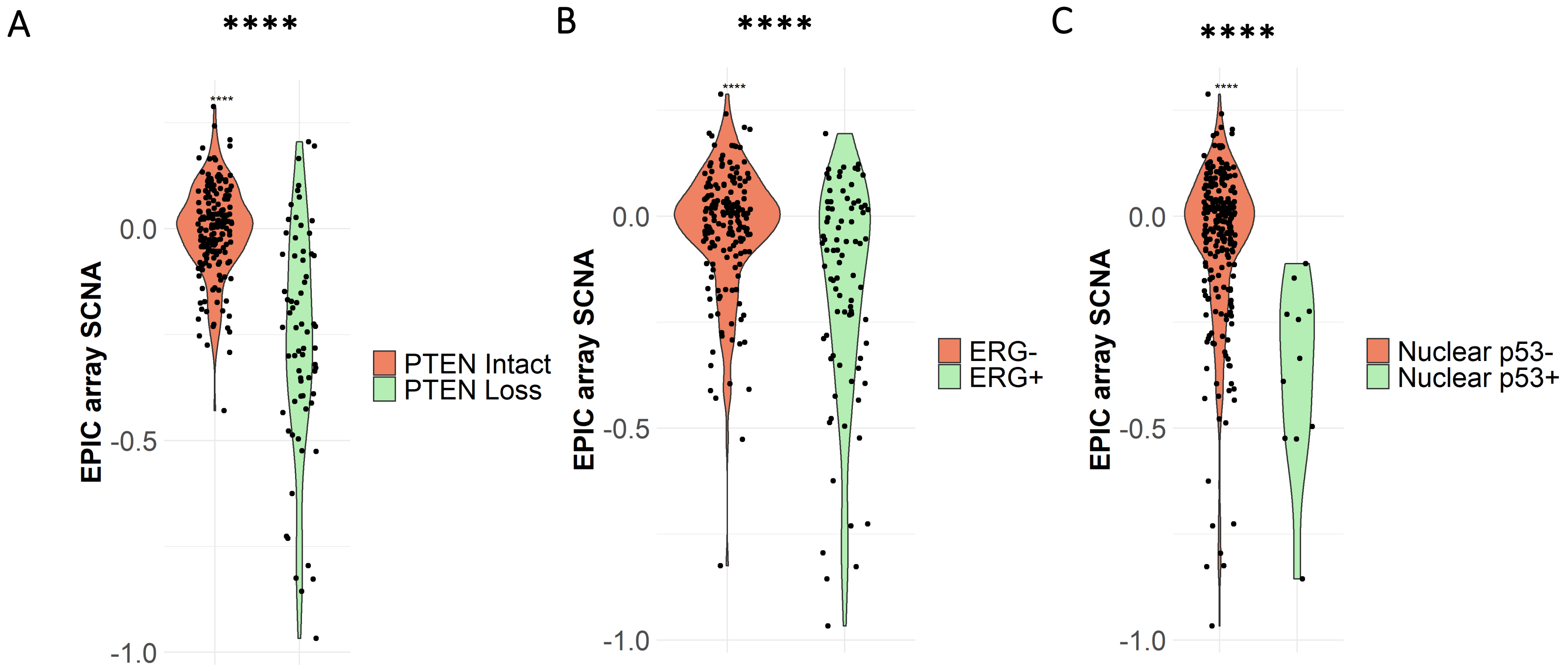

Supplementary Figure S6 – (A) Spearman correlation analysis showing a positive and significant association between PGA derived from the EPIC array data and PGA obtained via panel-based sequencing. Only a subset of BL patients (n=134) had previously published targeted sequencing. Our EPIC array copy number data was significantly associated with genomic changes evidenced by IHC. (B) PGA from EPIC was not associated with PTEN protein loss by IHC nor ERG positive by IHC (C), but was significantly increased in samples with nuclear accumulation of p53 on IHC (D).

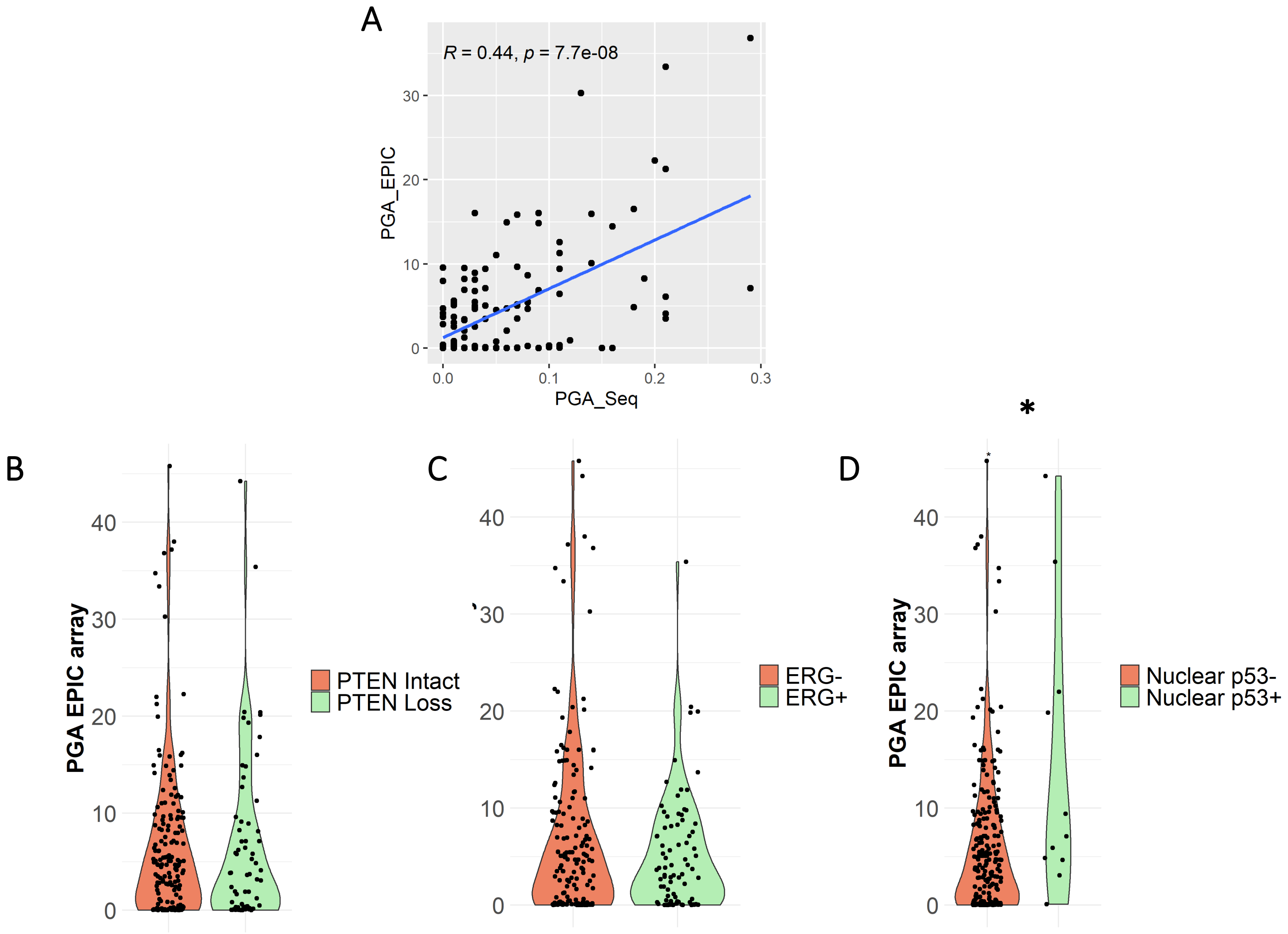

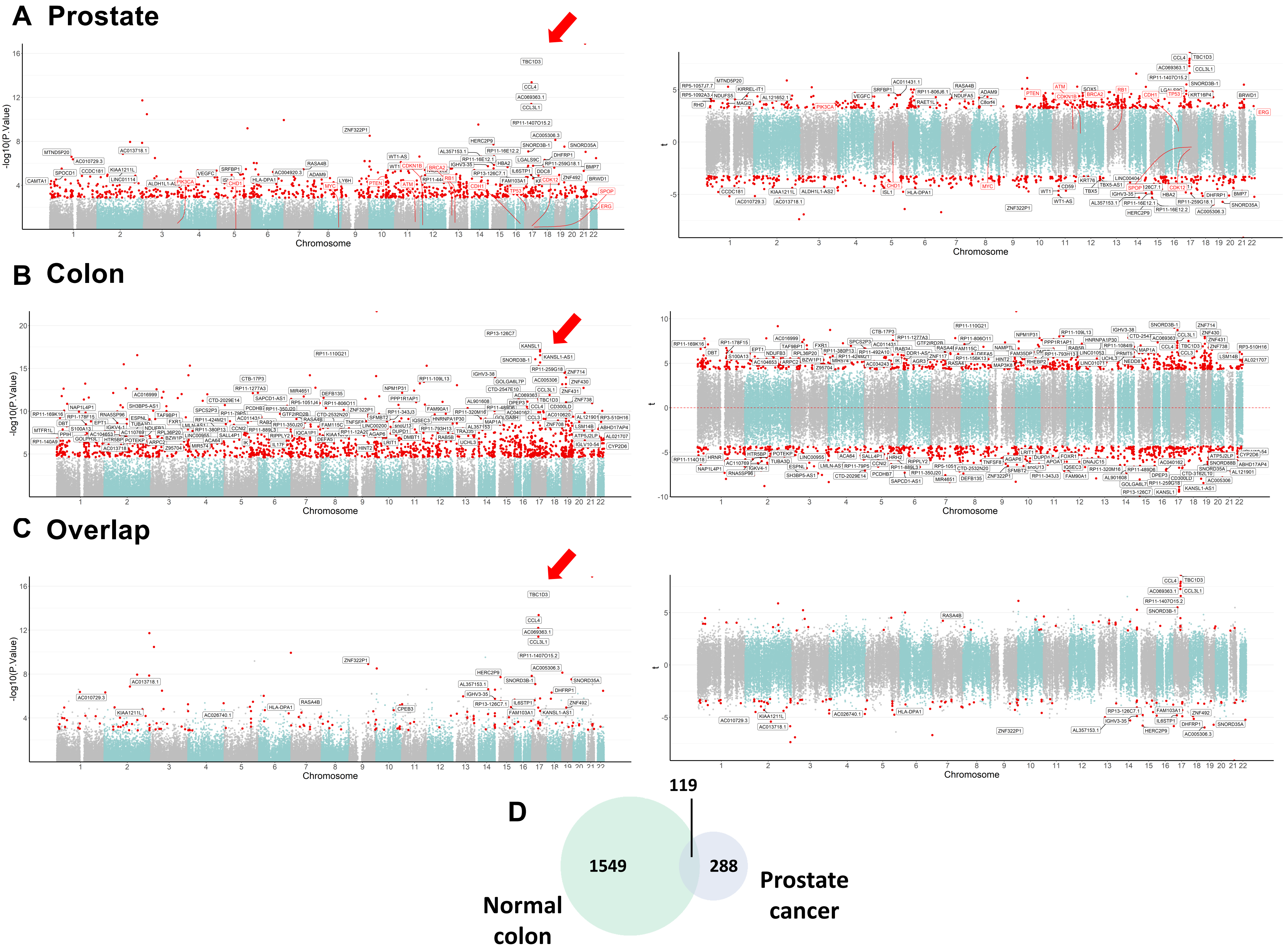
A Prostate
B Colon
C Overlap
D
-log10(P.Value)
t
Chromosome
119
1549
288
Normal colon
Prostate cancer

Supplementary Figure S7 – Copy number changes by self-identified race using Limma models in prostate tumor and benign colon tissue. p-values (left panel) and T-statistics estimated (right panel) are shown on the y-axis and are distributed by chromosome in the x-axis. Each dot represents a coding or non-coding locus. (A) Loci where copy number is associated with self-reported race in prostate cancer (number of significant loci = 407). Red dots show significant loci based on FDR-adjusted p-value (<0.1). Labels are shown for a subset of the coding genes with FDR-adjusted p-value <0.1. (B) Loci where copy number is associated with self-reported race for the normal colon cohort (number of significant loci = 1668). Red dots show significant loci based on FDR-adjusted p-value (<0.001). Labels are shown for genes with FDR-adjusted p-value <0.001. C) Overlap between significant loci from prostate cancer and colon models in A and B. Red dots show significant loci based on FDR-adjusted p-value (<0.1) in the prostate analysis overlapping with the genes from the colon analysis. For visualization purposes, a subset of genes are labeled with gene names. See **Supplementary Table S4** for the complete list of significant loci in prostate cancer.

Supplementary Figure S8 – (A) Chromosome 10 overview from the GLM model using percent African ancestry adjusted with PSA, Grade Group, and age. Each dot represents a coding or non-coding locus. Loci colors are shown based on their significance in the model. Genes shown in red are common cancer drivers found on chromosome 10. PTEN is more commonly lost in tumors from WH compared to BL patients. (B) Continuous sCNA score showing loci with significant alterations by self-reported race.

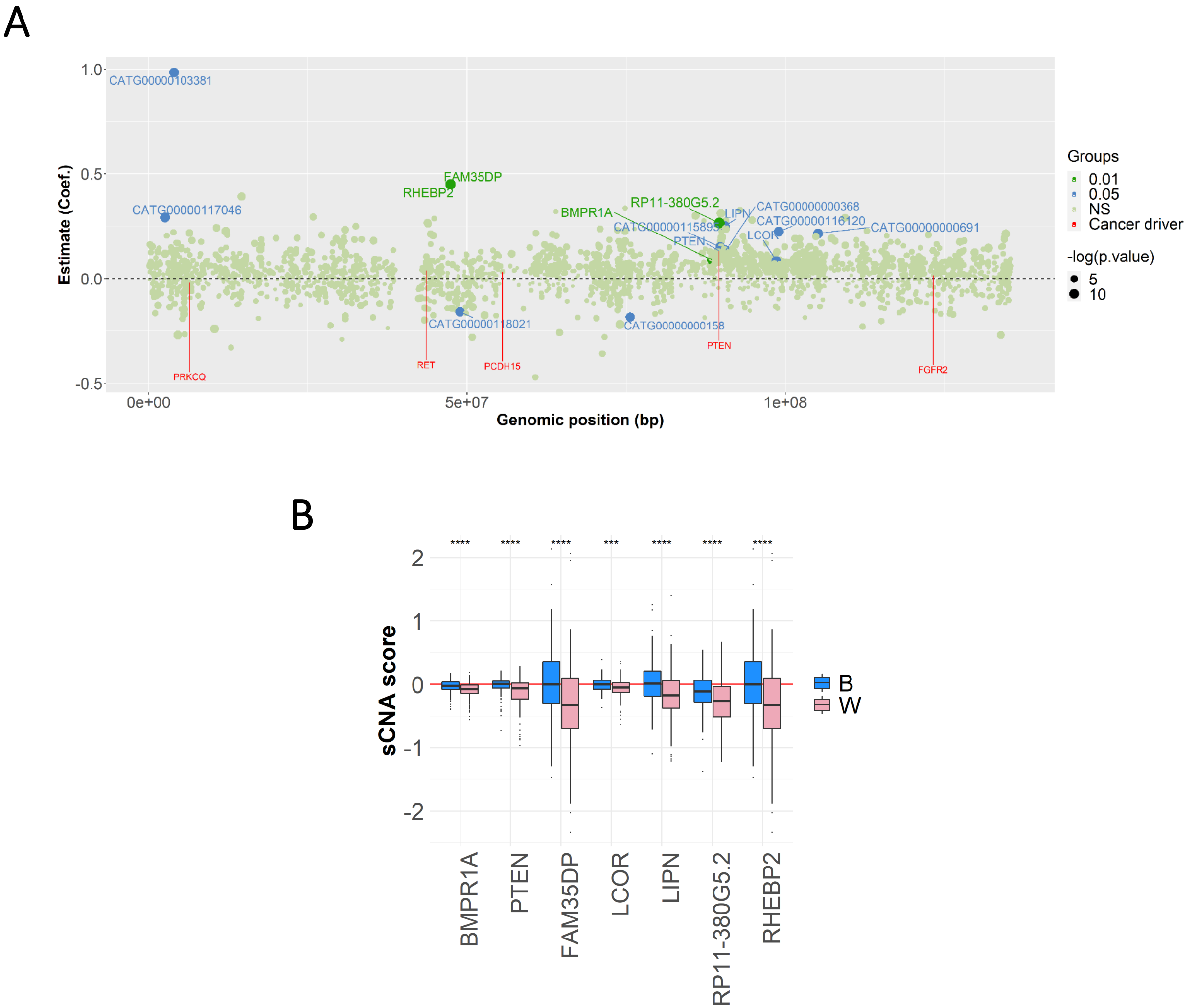

Supplementary Figure S9 - EPIC-array sCNA data for loci that are significantly associated with percent African ancestry adjusted with PSA, age, and Gleason grade from GLM models. Loci shown include only coding genes. For a summary statistic of each gene, see Table 2. **p<0.01; ***p<0.001; ****p<0.0001.

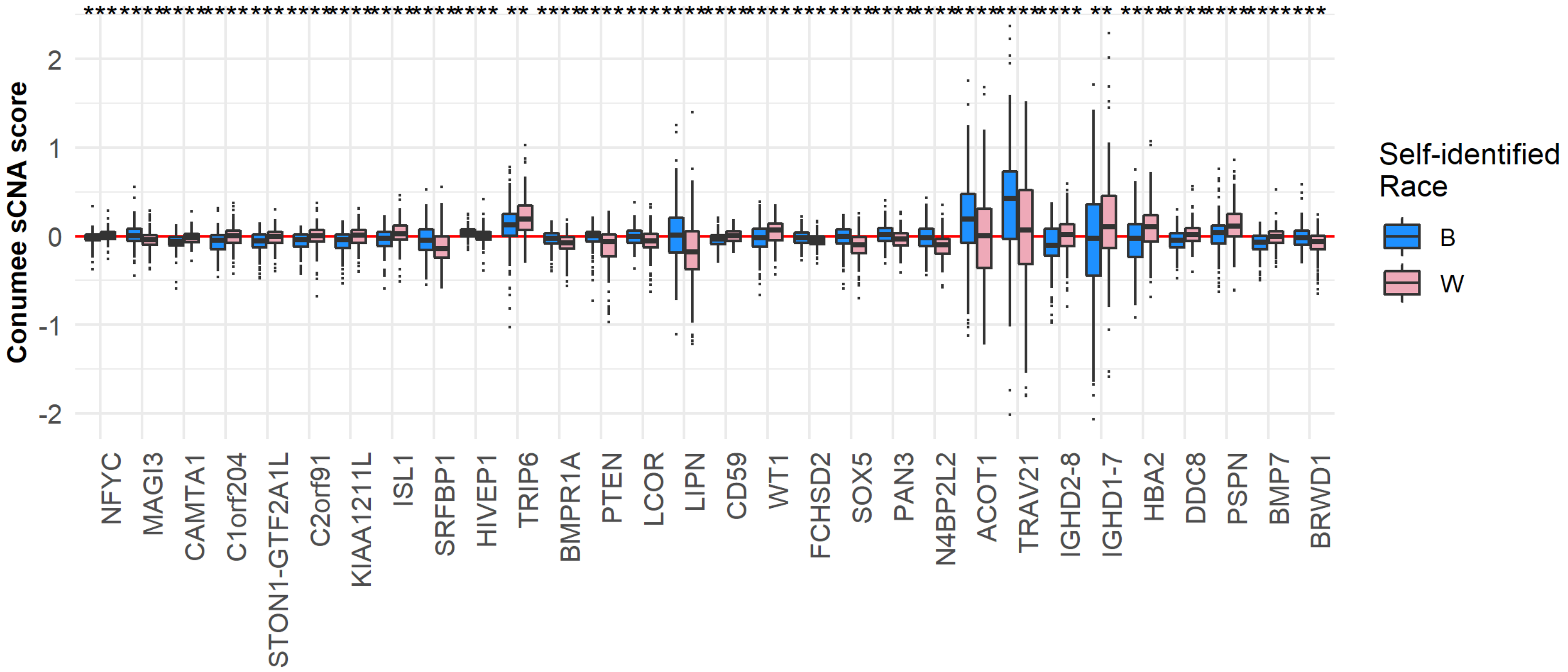

Supplementary Figure S10 - sCNA significantly associated with prostate cancer biochemical recurrence. Cox regression model adjusted with percent African ancestry, age, preoperative PSA, and tumor Grade Group (GG1 and GG2 vs. GG3, GG4, and GG5) was applied to all samples from our cohort (N=290) and showed significant associations between 8q gains and 10q and 12p losses with biochemical recurrence. (A) Hazard ratio shown by chromosome. Each dot represents a gene or lncRNA. Red dots show significant coding and non-coding loci based on FDR-adjusted p-value (<0.1). Coding genes with adjusted p-value <0.1 are labeled. See **Supplementary Table S8** for a list of all genes associated with biochemical recurrence.

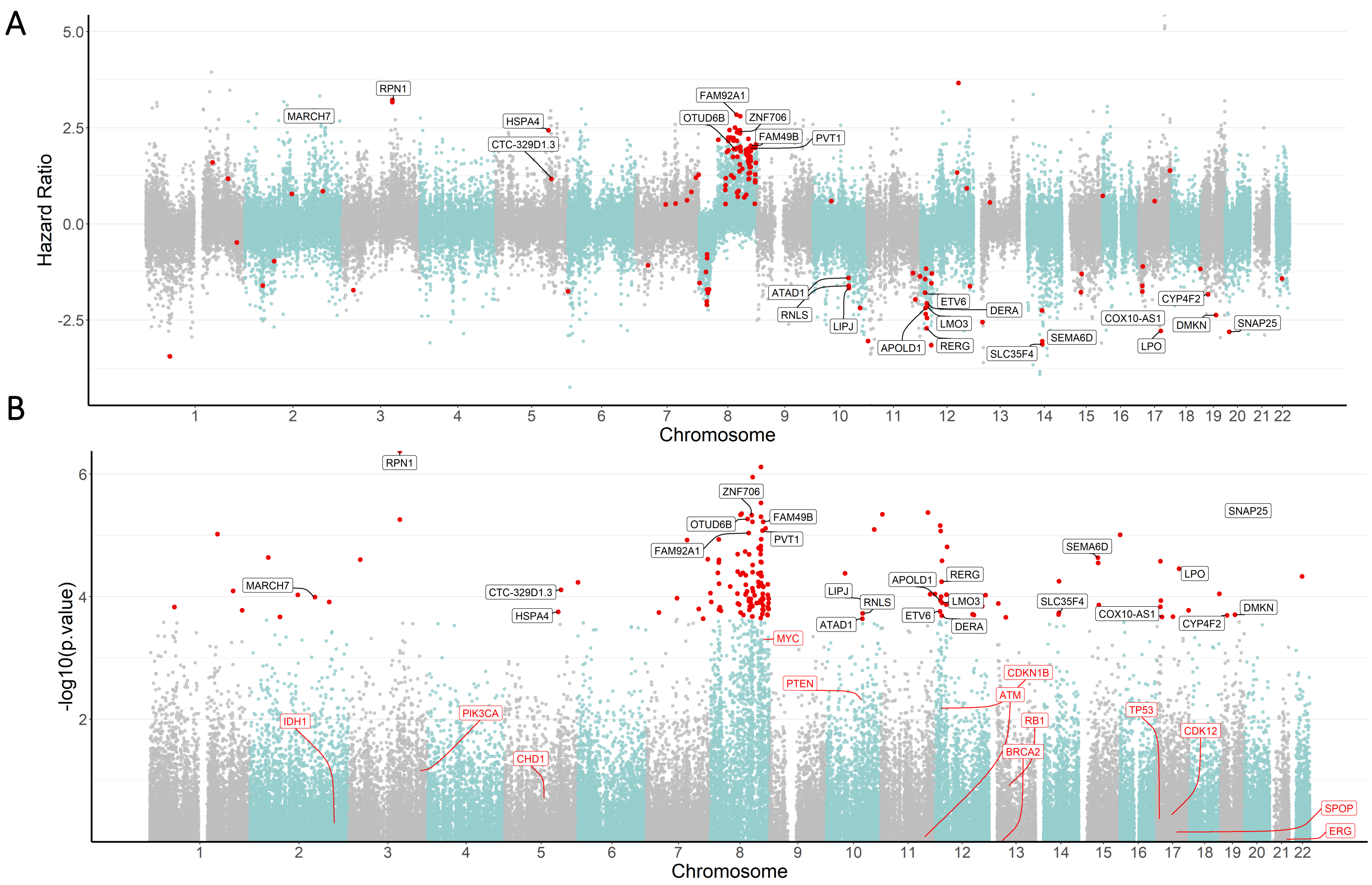

Supplementary Figure S11 - Correlation between tumor B-cell content and PGA by self-identified race. Panels A-C show correlations between CD19 cell content obtained from methylCIBERSORT and PGA for the Johns Hopkins prostate cancer cohort (n=249), BL patients (n=120), and WH patients (n=134), separately. Panels D-F show the correlations between previously published CD79+ cell density (via immunohistochemistry (IHC) (21) and PGA. Panels G-I show the correlations between CD19 cells content from methylCIBERSORT and PGA for the entire prostate cancer TCGA cohort (n=273), for BL patients (n=33), and for WH patients separately (n=222).

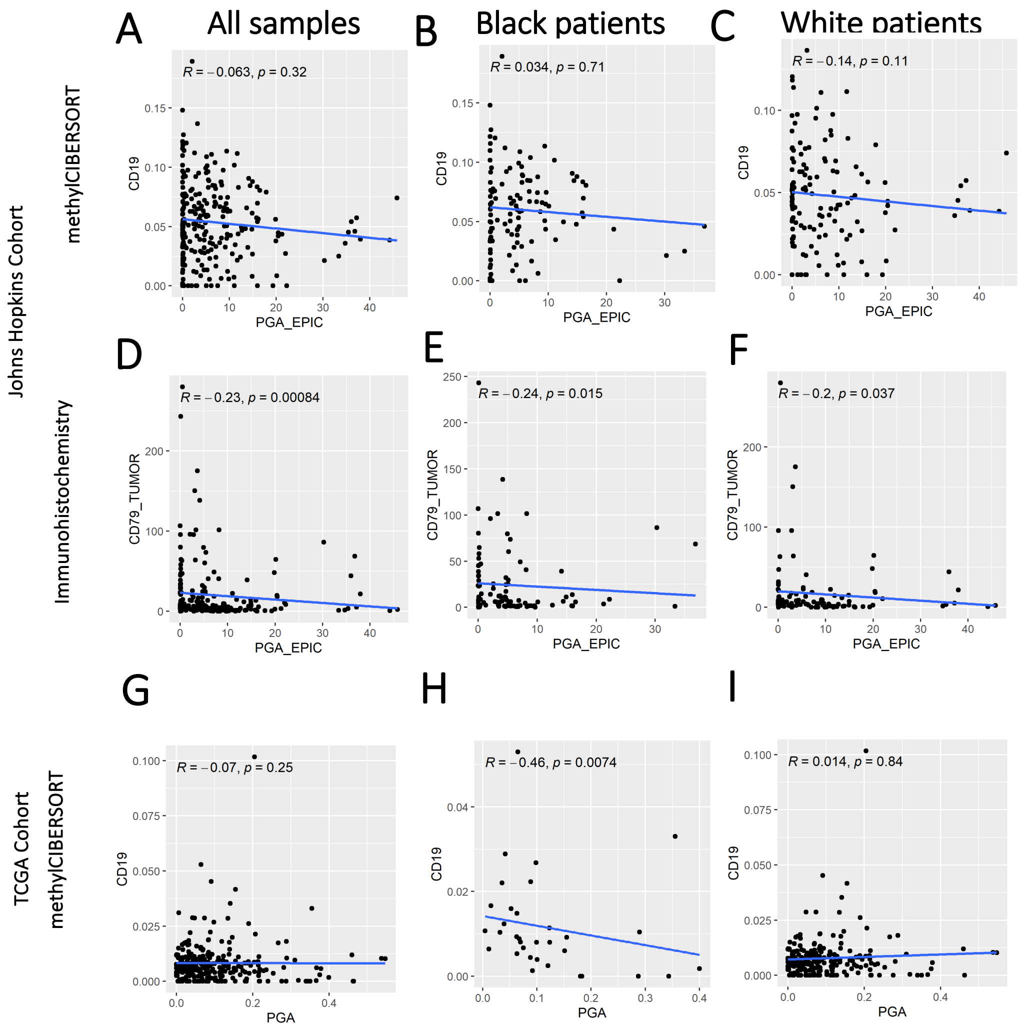

Supplementary Figure S12 - Correlation between T-cell content and PGA by self-identified race. Panels A-C show correlations between CD8 cell content obtained from methylCIBERSORT and PGA for the Johns Hopkins prostate cancer cohort (n=249), BL patients (n=120), and WH patients (n=134), separately. Panels D-F show the correlations between previously published CD8+ cell density via immunohistochemistry (IHC) (29) and PGA. Panels G-I show the correlations between CD8 cells content from methylCIBERSORT and PGA for the entire prostate cancer TCGA cohort (n=273), for BL patients (n=33), and for WH patients separately (n=222).

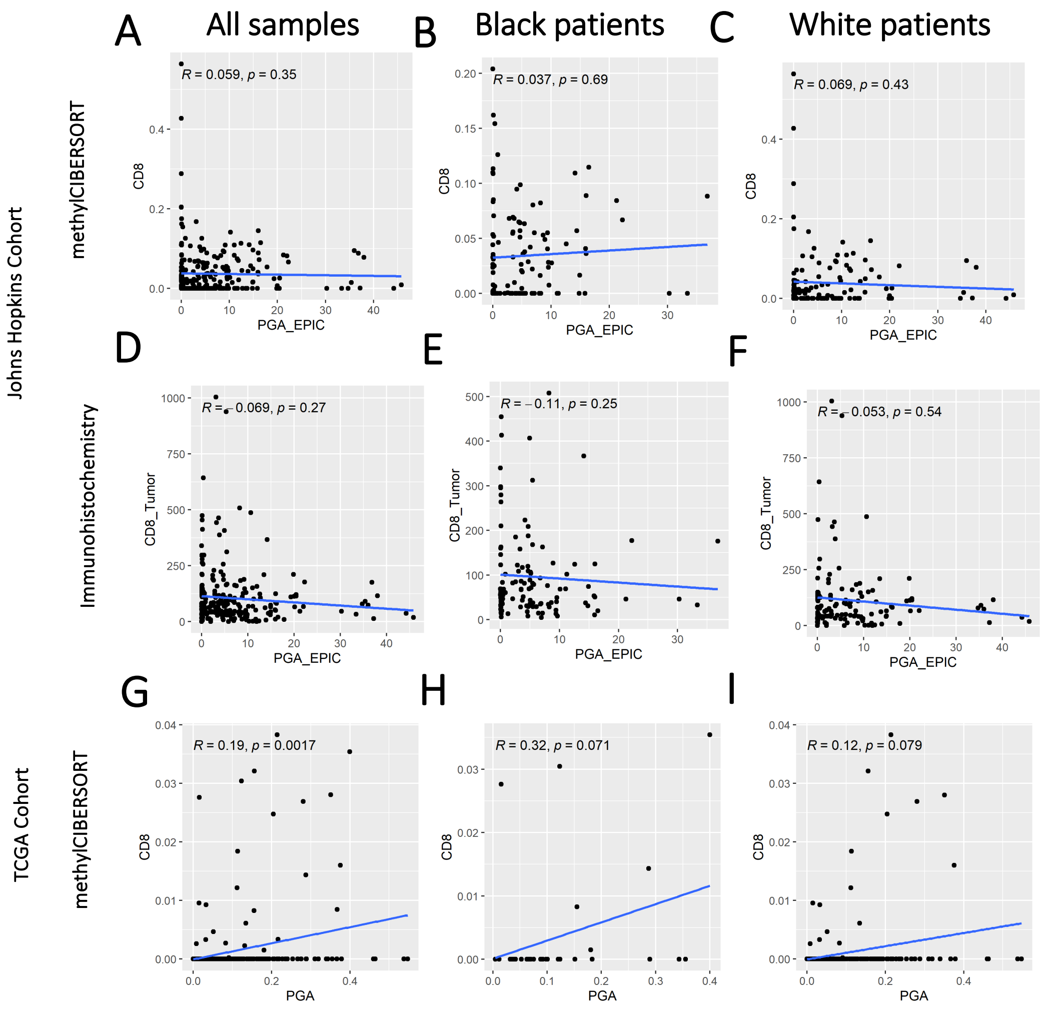

Supplementary Figure S13 - Correlation between macrophage content and PGA by self-identified race. Panels A-C show correlations between CD14 cell content obtained from methylCIBERSORT and PGA for the Johns Hopkins prostate cancer cohort (n=249), BL patients (n=120), and WH patients (n=134), separately. Panels D-F show the correlations between previously published CD163+ cell density via immunohistochemistry (IHC) and PGA. Panels G-I show the correlations between CD14 cells content from methylCIBERSORT and PGA for the entire prostate cancer TCGA cohort (n=273), for BL patients (n=33), and for WH patients separately (n=222).

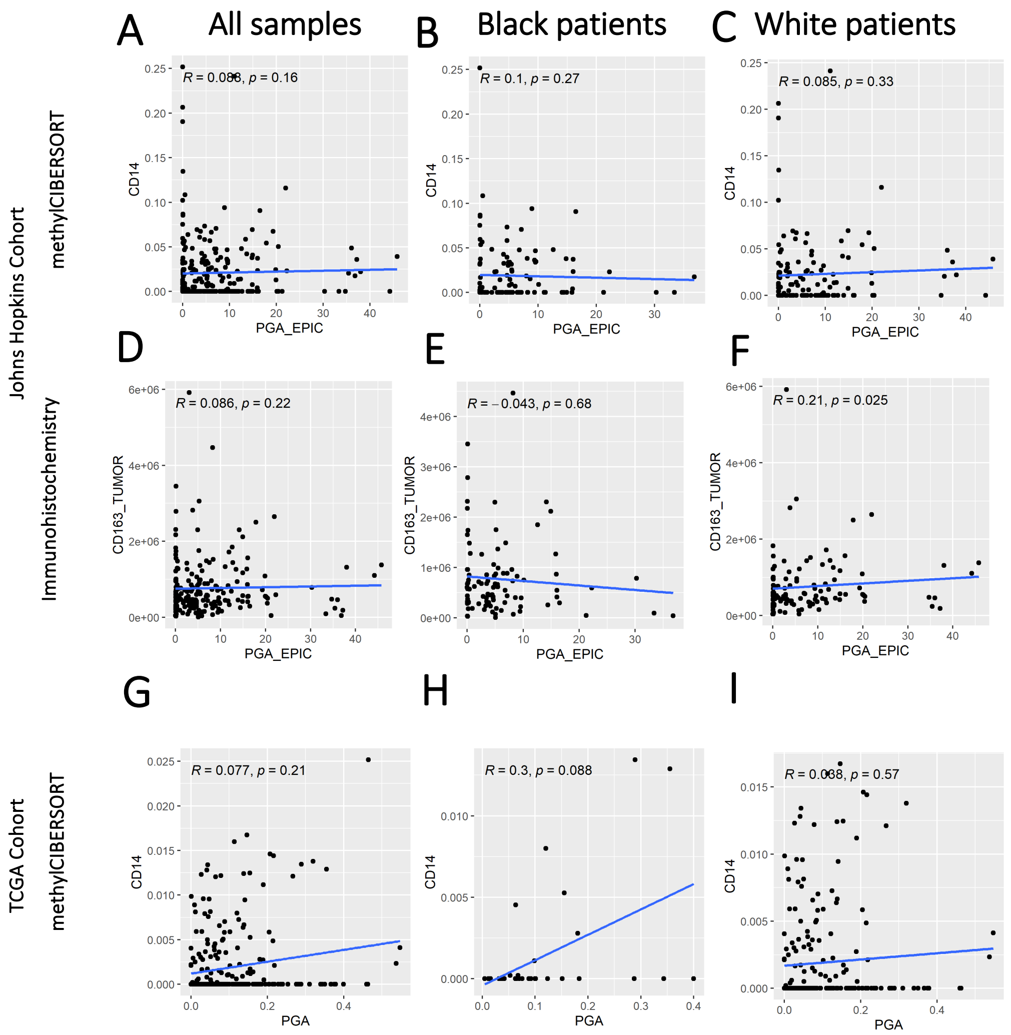

Supplementary Figure S14 - CC chemokine ligands gene expression (RNAseq z-scores) compared between self-identified Black and White patients from the TCGA cohort. **p<0.01; ***p<0.001.

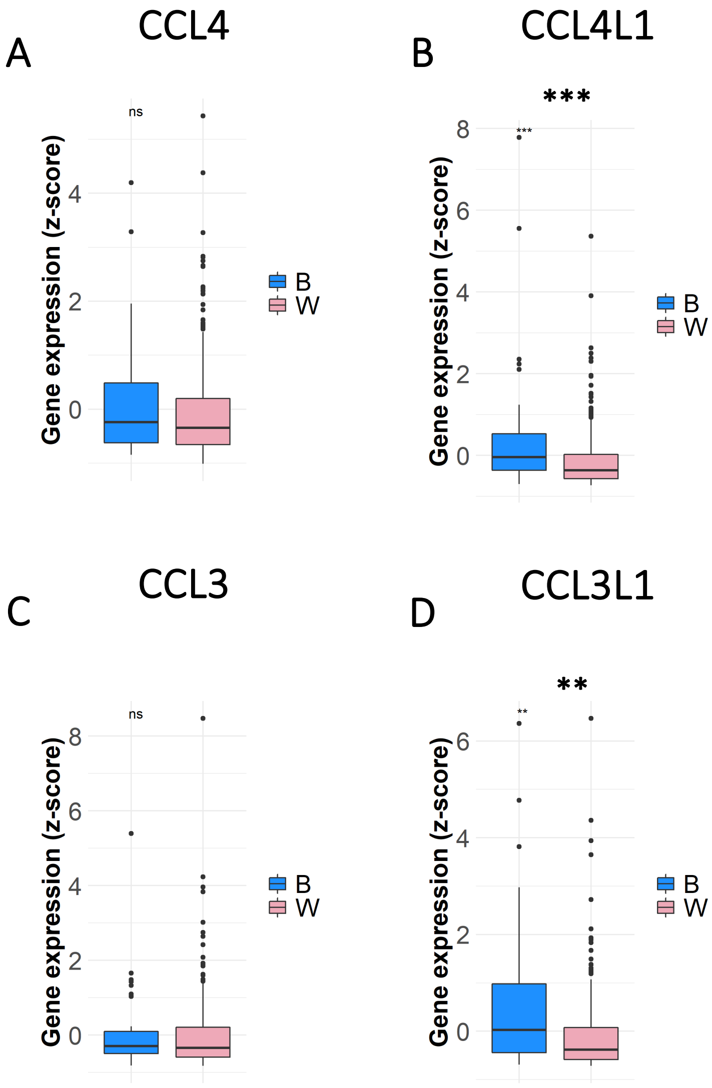

Supplementary Figure S15 - Summary of sample sizes for all methods employed in this study.

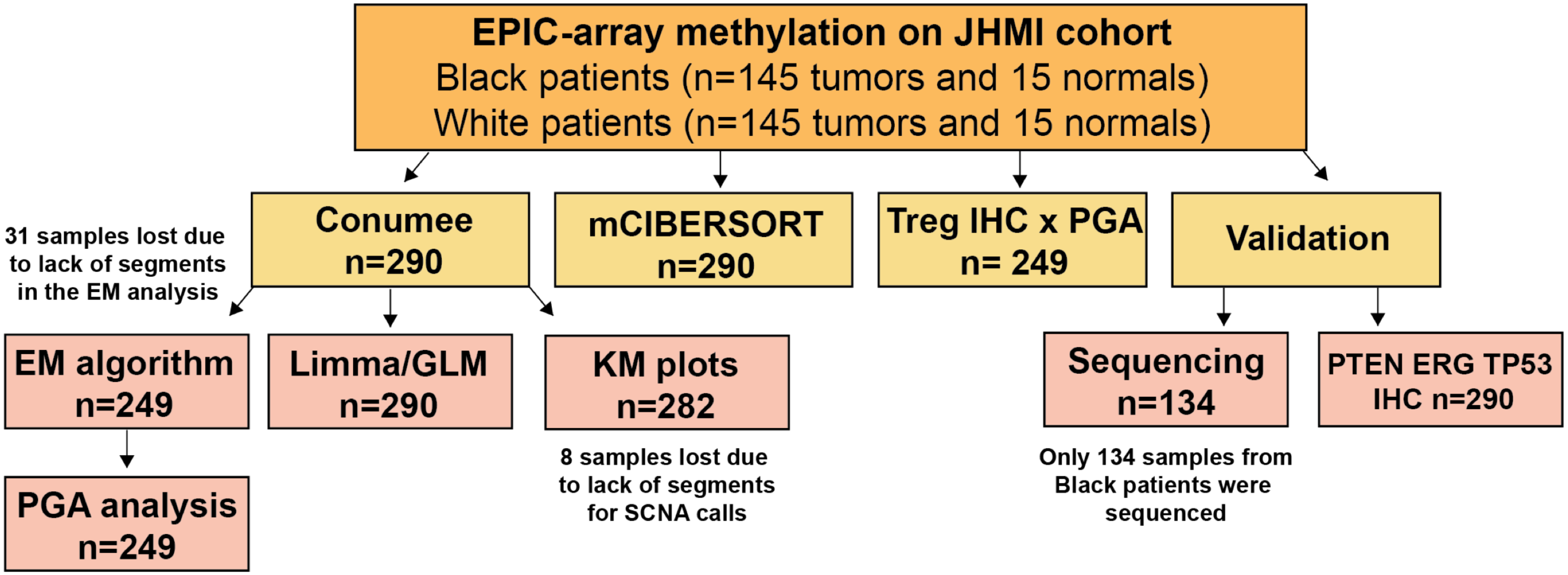

Supplement: Supplemental data [file jciinsight-8-162409-s114.pdf]
